# Influence of Docosahexaenoic and Eicosapentaenoic Acid Ratio and Temperature on the Growth Performance, Fatty Acid Profile, and Liver Morphology of Dusky Grouper (*Epinephelus marginatus*) (Teleostei: Serranidae) Juveniles

**DOI:** 10.3390/ani13203212

**Published:** 2023-10-14

**Authors:** Ethiene Fernandes de Oliveira, Bruno Cavalheiro Araújo, Victor Hugo Marques, Paulo Henrique de Mello, Renata Guimarães Moreira, Renato Massaaki Honji

**Affiliations:** 1 Departamento de Fisiologia, Instituto de Biociências, Universidade de São Paulo (IB/USP), Rua do Matão, trav. 14, nº 321, São Paulo 05508-090, SP, Brazil; feroliver.thi@gmail.com (E.F.d.O.); renata.fish@gmail.com (R.G.M.); 2 Núcleo Integrado de Biotecnologia, Universidade de Mogi das Cruzes (UMC), Mogi das Cruzes 08701-970, SP, Brazil; brubiol1@gmail.com; 3 Beacon Development, King Abdullah University of Science and Technology, Thuwal 23955-6900, Saudi Arabia; paulombio@gmail.com; 4 Centro de Biologia Marinha, Universidade de São Paulo (CEBIMar/USP), Rodovia Manoel Hipólito do Rego, km 131,5, São Sebastião 11612-109, SP, Brazil

**Keywords:** brain, DHA, EPA, eyes, lipid, muscle

## Abstract

**Simple Summary:**

Aquaculture production is the fastest-growing activity in the agriculture sector worldwide. Nutritional studies are always in demand because fish feeding is one of the main points that need to be solved in modern marine aquaculture since feeding is responsible for almost 70% of the total cost of production. Nutrition is still considered a challenging part of dusky grouper aquaculture due to the high costs of fish meal and fish oil, which need to be incorporated into aquaculture diets to satisfy the nutritional requirements of marine species. We evaluated the effects of fatty acids, provided as different lipid classes in diets, to provide new insights about dusky grouper juvenile nutrition, and we also demonstrated the influence of temperature on lipid metabolism. The main contribution of our study was to show that temperature influences lipid metabolism and that vegetable oils can replace fish oil, but only if the diet is supplemented with different lipid classes, mainly with omega-3. Another important outcome from the study was to show that increasing omega-3 levels in the diet does not increase its content proportionally in animal tissues, information that is important from an economic perspective. Therefore, our results provided valuable knowledge of dusky grouper nutrition and physiology, helping to solve one of the bottlenecks in modern aquaculture.

**Abstract:**

An 8-week trial was performed to evaluate the influence of docosahexaenoic (DHA) and eicosapentaenoic acid (EPA) ratios and temperature on the fatty acid (FA) metabolism and liver morphology of *Epinephelus marginatus*. A basal diet was manufactured, and DHA (D1:0.5%; D2:0.75%; D3:1%; and D4:1.5%) and EPA (D1:1.0%; D2:0.75%; D3:0.5%; and D4:0%) levels were added. *E. marginatus* were kept in twelve tanks with a lower temperature (LT, 23.17 ± 0.12 °C) and twelve with a higher temperature (HT, 28.63 ± 0.06 °C). The DHA/EPA ratio did not affect performance, regardless of the temperature, but the animals fed with the D4 diet showed better growth at HT. A higher lipid deposition and a large lipid vacuole area in the hepatocytes at HT were observed, regardless of the diet. Triacylglycerol (TG) in reflected the diet FA profile mainly in the muscle and liver, whereas the phospholipid (PL) was less influenced by the diet. The same DHA content in the TG fraction of muscle (D3 and D4) were observed at LT; however, only the DHA/EPA ratio of D4 could be differentially stored in the TG fraction of muscle (HT). Monounsaturated and polyunsaturated FA increased in the PL of the eyes at HT, whereas saturated FA was reduced in the TG and PL fractions at LT. These results evidence the importance of temperature and a balanced DHA/EPA ratio in the diet of marine fish.

## 1. Introduction

The dusky grouper (*Epinephelus marginatus*) is a marine teleost in a vulnerable condition on the Red List of the International Union for Nature Conservation (IUNC) [1,2]. Late sexual maturation [1,3,4,5], overfishing, and especially the lack of adequate and specific management [2] are factors that intensify the species’ natural conservation status. The consumer demand for this species is mainly due to the positive commercial characteristics presented, such as the large size, and fillets with great texture, color, and flavor. In addition to these characteristics, *E. marginatus* is management-resistant, making it a promising species for Brazilian aquaculture, enabling their production in captivity and reducing extractive fishing [1,4,5,6,7,8,9,10]. However, information related to the physiology/nutrition/aquaculture of this species is scarce in the literature, as species–specific studies are necessary to assist their rearing in captivity, mainly on topics related to the reduction of the total cost of production.

Captive production emerges as a viable alternative for the conservation of *E. marginatus*; however, as observed for other teleost species commonly cultivated, the production of specific diets considering the physiological characteristics of the species can be the main limiting factor for the increase in the production of this species, since most of the production costs (approximately 70% of the total cost) are directly related to nutrition [11]. The high cost of production is directly linked to use of ingredients such as fish meal and oil, which are expensive ingredients and unavailable on the world market. Additionally, it is highly impactful on the environment, as part of these inputs are produced from natural fishing stocks [12,13,14,15]. In recent decades, there has been a striving effort to replace, mainly, fish oil (FO) with vegetable oils (VOs), such as soybean, coconut, and olive oil. However, the main limitation for the use of these alternative lipid sources in marine fish nutrition is related to their low levels of long-chain polyunsaturated fatty acids of the n-3 series (LC-PUFA n-3) [14,16,17], since, in general, marine fish have limited capacity to synthesize these fatty acids from their precursors, which are acquired exclusively through diet [6,15,18]. In fact, the influence of LC-PUFA n-3 on the physiology of *E. marginatus* does not exist in the specialized literature.

Docosahexaenoic acid (DHA, 22:6n-3) and eicosapentaenoic acid (EPA, 20:5n-3) are the physiologically most relevant LC-PUFAs for marine fish. These fatty acids (FAs) play fundamental roles in marine fish, and adequate levels of these substrates are essential for proper development since the inadequate incorporation of these FAs in diets can negatively influence important physiological processes, such as tissue development, the prevention of various pathologies, and the balance of the immune system, in addition to impacting, in general, the lipid metabolism of the target species [13,14,15,19,20]. Additionally, the lack of these ingredients in diets can result in a low-quality final product, generating a fillet with low nutritional value, as these FAs are relevant to human nutrition [13,16]. In addition to the adequate balance of DHA and EPA in diet, factors such as temperature can reduce the quality of the final product, since temperature can affect nutrient intake and the way in which fish use the energy acquired through diet, since most teleosts are ectotherms [13,15,17]. Furthermore, DHA and EPA are LC-PUFAs present in biological membranes, and, because they have a specific chemical structure, they constitute fluidity [15]. Fluidity is an important factor for cell membranes, as the greater the fluidity, the more correctly and quickly intra- and extracellular communications are carried out, facilitating the biological processes mentioned above. It is important to highlight that temperature can affect the fluidity of membranes in different ways: (1) low temperature = ordered liquid state, which is the semi-solid state in the bilayer; and (2) high temperatures = disordered liquid, which is the most fluid state in the bilayer) [13,15]. Additionally, the fluidity of membranes varies according to the different physiological processes mentioned [13,14,15,19,20].

In addition to the important properties previously mentioned, DHA and EPA are among the main constituents of phospholipids, which are essential lipids that control the fluidity of the biological membranes of important tissues such as the nervous and visual ones, being essential for the structuring and functioning of cells in these and other tissues [13,21,22]. Phospholipids are known to be structural components of biological membranes that respond to changes in temperature [23,24]. Therefore, the FA composition of this lipid class is relevant in rearing marine fish, since most cultivated species are exposed, at some period of the production cycle, to suboptimal temperatures. As, normally, the composition of the diet offered to farmed fish together with abiotic factors, especially temperature, directly influence the composition of tissues and, consequently, the growth performance, health, and quality of the final product, investigations focused on this interface on species with high productive potential and which are ecologically pressure, such as as *E. marginatus*, are considered essential to improve its productivity.

Considering all the information previously described, the commercial production of *E. marginatus* can be considered a viable alternative for reducing the pressure on the species’ natural stocks; however, nutrition is still seen as the main barrier to large-scale production. Given the importance of DHA, EPA, and the relationship of dietary lipid composition with water temperature in the physiology and growth performance of marine fish, and, due to the lack of information related to these aspects in the nutrition of *E. marginatus*, this study proposed to investigate whether the effects of different DHA/EPA ratios can interfere with the growth performance and FA composition (in triacylglycerol and phospholipids) of the lipolytic and lipogenic tissues of *E. marginatus* juveniles. Additionally, considering the role of the liver as an important lipid deposit in the teleost, the morphology of this tissue was qualitatively evaluated.

## 2. Materials and Methods

### 2.1. Diet Manufacturing

The diet formulation and proximate composition are presented in Table 1, while the FA profile of the experimental diets and oils used for manufacturing are shown in Table 2. The fish and poultry meals used were previously defatted by immersion and washing in hexane (fish and poultry meals 3:1 solution). Four isoproteic, isolipidic, and isoenergetic diets were formulated containing different lipid sources. The experimental diets were formulated using a lipid blend containing coconut oil (source of saturated fatty acids—SFA), olive oil (source of monounsaturated fatty acids—MUFA), concentrated arachidonic acid (ARA) oil, and different inclusion levels of DHA and EPA concentrated oil. Each diet consisted of one dietary treatment, including equal levels of coconut, olive, and ARA oils, varying in the levels of EPA and DHA concentrated oils:

- D1: 1.00% EPA and 0.50% DHA;
- D2: 0.75% EPA and 0.75% DHA;
- D3: 0.50% EPA and 1.00% DHA;
- D4: 0.00% EPA and 1.50% DHA.

The diets were manufactured, and the experimental-blend oils were vacuum-coated to the pellets during the mixing process. A vacuum pump was attached to evacuate the air, and the lipid-blend oils were infused into the pellets when the atmospheric pressure was re-equilibrated. This method was performed according to previous studies with other marine teleost species [17,25]. After the vacuum-coated process, the pellets were dried in forced air circulation stoves for 24 h, and, subsequently, stored at −20 °C until the beginning of the experiment. The proximate composition of the experimental diets was analyzed in triplicate according to the methodology implemented by [26] (Table 1).

### 2.2. Experimental Design, Fish Handling, Productive Performance, and Biological Index

*Epinephelus marginatus* juveniles were obtained from *Redemar Alevinos* (Ilhabela, North Coast of São Paulo State, Brazil) and transported to the Marine Experimental Facilities at the Centre of Marine Biology of the University of São Paulo (CEBIMar/USP), also located in São Sebastião, North Coast of São Paulo State, Brazil. The fish were maintained for two weeks in a 10,000 L tank and hand-fed twice a day (08:00 and 17:00 h) until apparent satiation with a commercial marine fish diet (Guabipirá, Guabi Nutrição e Saúde Animal SA, SP, Brazil). After that, the animals were transferred to twenty-four 500L experimental tanks in a recirculation system equipped with a UV, biological filter, mechanical filter, pump, and water heater. In this study, two temperatures were considered. Twelve tanks were maintained at an average temperature of 28.63 ± 0.06 °C (triplicate for each experimental diet, namely, higher temperature—HT), and another twelve tanks were kept at an average temperature of 23.17 ± 0.12 °C (triplicate for each experimental diet, namely, lower temperature—LT). Seawater was supplied continuously at a flow of ~10 L min^−1^ (both temperatures), the water salinity was 31 PSU (both temperatures), the dissolved oxygen was 4.55 ± 0.06 mg L^−1^ (HT) and 5.30 ± 0.03 mg L^−1^ (LT), the total ammonia nitrogen (TAN) was lower than 0.05mg L^−1^ (both temperatures), the nitrite level was lower than 0.05 mg L^−1^ (both temperatures), and the nitrate level was lower than 1.20 mg L^−1^ (both temperatures). The water temperature and dissolved oxygen were measured daily (YSI model 55, YSI Inc., Yellow Springs, OH, USA), and the total ammonia nitrogen, nitrite and nitrate levels were measured every three days (API test kits, Mars Fishcare Inc., Chalfont, PA, USA).

At the beginning of the experiment, each tank held 9 fish with an average body mass of 38.85 ± 0.70 g (*n* = 9 animals per tank; *n* = 27 animals per experimental diet group; *n* = 108 animals per temperature group (HT and LT); *n* = 216 total animals used in this study). Additionally, ten animals from the initial stock were anesthetized with 0.1% benzocaine (*ethyl-p-aminobenzoate*) and samples of liver, muscle, the whole brain, and both eyes were immediately frozen in liquid nitrogen and maintained at −80 °C until analyses. During the nutritional experiment, fish were hand-fed at 08:00 and 17:00 h with the different diets at a rate of 3% biomass/day, for eight weeks (56 days), and this rate was adjusted every fifteen days. At the end of the experiment trial, all animals were anesthetized with 0.1% benzocaine, and their biometrical parameters were recorded. Then, three fish per tank (nine fish/experimental diets at each temperature) were euthanized and dissected. Thereafter, the liver was quickly removed and weighed to calculate the hepatosomatic index (HIS). Samples of liver and muscle, the whole brain, and both eyes were frozen in liquid nitrogen and maintained at −80 °C until analyses. Other samples of the liver were fixed in formalin solution (4%) for 24 h, and then preserved in 70% ethanol until the histological analyses.

The productive performance and biological index were evaluated using the following parameter metrics:

Weight gained (WG, g) = W_f_ − W_i_;

Daily gain rate (DGR, g/day) = (W_f_ − W_i_)/t;

Specific growth rate (SGR, %d) = (LnW_f_ − LnW_i_) × 100/t;

Hepatosomatic index (HSI, %) = 100 × (liver weight/W_f_);

Survival rate (SR, %) = N_f_ × 100/N_i_.

where:

W_f_ and W_i_ = final (W_f_) and initial (W_i_) fish weight, respectively;

N_f_ and N_i_ = final (N_f_) and initial (N_i_) number of fish, respectively;

t = duration of experiment, in days.

Fish handling and the procedures used in the sampling of the animals were performed in accordance with the Animal Ethics Committee of the Institute of Biosciences, University of São Paulo (IB/USP) (CEUA protocol 356/2019).

### 2.3. Total Lipids and Fatty Acid Profile Analyses

The total lipids of the experimental diets and tissues were extracted with a mixture of chloroform, methanol, and water (respectively, 2:1:0.5) following [27] modified by [28] for aquatic organisms. Next, the total lipid content was quantified by the colorimetric method of [29], using cod liver oil (cod liver oil fatty acid methyl esters, Sigma Diagnostics, St. Louis, MO, USA) to yield the standard curve. The extracted lipids from the liver, muscle, brain, and eyes were separated into triacylglycerol (TG) and phospholipid (PL) fractions by thin-layer chromatography using an activated silica column [30]. Triacylglycerol and phospholipid fractions of liver, muscle, brain, and eyes were methylated with acetyl chloride (5% HCl in methanol) and converted into fatty acid methyl-esters [31].

Fatty acid methyl-ester (FAME) analysis was carried out in a Varian gas chromatograph (GC; Model 3900, Walnut Creek, CA, USA) coupled with a flame ionization detector (FID) and a CP-8410 autosampler. The capillary column for analyzing the FA was a CP Wax (30 m length × 0.25 mm inner diameter with 0.25 μm thickness) using hydrogen as the carrier gas at a linear velocity of 22 cm/s. The following temperature program was used: 170 °C for 1 min, followed by a ramp of 2.5 °C/min until reaching 240 °C, and a final waiting time of 5 min, totaling 31 min of running. In the injector, the temperature was 250 °C and, in the flame ionization detector (FID), the temperature was 260 °C. Using known methyl-ester standard FAME (Supelco, 37 components; Sigma-Aldrich, St. Louis, MO, USA; Mixture, Me93; Larodan and Qualmix, PUFA fish M, Menhaden Oil, Larodan), FAs were identified by comparison with retention time. Data were presented as a percentage of the total FAME based on the peak area analyzed.

### 2.4. Liver Histology Analysis

Liver samples were fixed in formalin solution (4%) for 24 h, dehydrated through an ascending series of increasing concentrations of ethanol, cleared in dimethylbenzene solution (xylene), and embedded in Paraplast^®^ according to routine histological procedures for the preparation of liver permanent histological cross-sections [32]. Liver sections (5 μm thick) were obtained using a microtome (Leica Histo Core Auto Cut), equipped with disposable blades and mounted on Poly-_L_-Lysine-solution-coated slides. The slides were stained with hematoxylin–eosin, examined, and documented using, respectively, a light microscope (Leica DM1000 LED light microscope) coupled to a camera (Leica MC170HD photography camera) and a computerized image capture system (Leica LAS Interactive Measurements; 1260 pixels by 960 pixels) to evaluate the hepatocyte morphology and to measure the average area of these cells.

For the hepatocyte morphological analyses, the average area of these cells was considered. Within this purpose, fifteen cells per section were quantified, and three sections per sample (*n* = 3 fish/tank; *n* = 45 cells/fish measured; 135 cells per experimental group) with 100 μm distance between each section (totaling 300 μm distance from the first section to the last part). The “LAS Interactive Measurements” was also used to obtain the measurements of the average hepatocyte area (in μm^2^) according to previous studies performed with teleost and invertebrate’s species, such as *Rachycentron canadum* [17,25], *Morone saxatilis* [33], *Steindachneridion parahybae* [34,35], and *Litopenaeus vannamei* [36].

### 2.5. Statistical Analyses

A descriptive analysis was performed, and all values were expressed as the mean ± standard error of the mean (M ± SEM). Data normality and homogeneity of variance were analyzed, and comparisons between experimental groups were performed using two-way ANOVA, considering diet and temperature as factors, and followed by Tukey’s HSD tests using the statistical software SigmaStat for Windows ver. 3.10 (Systat Software, San Jose, CA, USA). Statistical difference was used considering a significance level of 5% (*p* < 0.05).

The ratio between tissue FA composition and dietary FA composition (tissue/diet) in liver and muscle TG was calculated as follows: the mean (%) of a specific FA divided by the mean (%) of the same FA in each tissue. The dotted line in the graphics indicates higher (above 1.0), lower (below 1.0), or equal levels (1.0) of a specific FA deposited in the tissues relating to the same FA in the diet. This ratio was calculated for muscle and liver tissues (and not for eyes and brain), since they are FA storage tissues and of interest (mainly muscle) for aquaculture, according to previous studies [25].

## 3. Results

### 3.1. Biological Indices and Productive Performance

The results related to the performance and biological index were presented in Table 3. No mortality was observed during the trial in any of the experimental groups. No significant differences were observed in the HSI between fish from different treatments at either temperature. Fish from D4-HT showed higher final weight, weight gain (WG), and daily gain rate (DGR) than those from the D4-LT group. Additionally, D4-HT fish also presented a significantly higher final weight than fish from the D2-HT group.

### 3.2. Tissues’ Total Lipids and Fatty Acid Profile

The total lipid concentrations of dusky grouper tissues were presented in Table 4. In muscle, the single significant change observed was the higher lipid content in fish from the D4-LT group compared to those from D4-HT. In liver, regardless of diet, it was possible to observe a greater lipid deposition in fish at a higher temperature. Additionally, fish from the D2-LT group had a lower lipid content in the liver compared with those from the other nutritional treatments, at the same temperature. The lipid concentration in the brain was higher in fish from the D1-LT and D3-LT groups compared to fish from the D1-HT and D3-HT groups, respectively. No significant changes were observed in lipid content in the eyes of fish from different experimental groups and kept at different temperatures.

Due to the massive FA database obtained in this study, we described in this section the results obtained in SFA, MUFA, PUFA, DHA, and EPA, and all data were presented in Table 5, Table 6, Table 7, Table 8, Table 9, Table 10, Table 11 and Table 12.

The triacylglycerol (TG) FA composition of the muscle, liver, eyes, and brain were presented in Table 5, Table 7, Table 9, and Table 11, respectively, while the phospholipid (PL) composition of these same tissues was presented in Table 6, Table 8, Table 10, and Table 12, respectively. Generally, the total SFA levels in muscle (Table 5 and Table 6) and liver (Table 7 and Table 8) were similar regardless of dietary treatment. However, fish maintained at the higher water temperature presented higher levels of SFA in the TG fraction of muscle when they were fed the diets D2 and D4, compared with the lower temperature (Table 5). In the brain and in the eyes, SFA was mainly influenced by the levels of 18:0. SFA was higher in the TG fraction of the eyes of fish fed with diets D1, D2, and D4 exposed to the lower temperature compared to the higher one, while fish from the D4 treatment had a higher SFA content in the TG fraction than fish from D1, when both groups were maintained at the lower temperature (Table 9). In the PL fraction, fish from treatments D3 and D4 presented a higher SFA content in the eyes when maintained at the lower temperature compared to the higher one (Table 10). The SFA profile in the brain did not change in the PL fraction (Table 12) and was higher in the TG fraction of fish from the D1 treatment maintained at the higher temperature, when compared with the other treatments (D1, D2, and D3) in the higher temperature and compared to fish from D1 maintained at the lower temperature (Table 11).

Significant changes were observed in the total MUFA of TG and PL, mainly influenced by changes in the 18:1n-9 percentage. The MUFA content did not change in the liver (Table 7 and Table 8), while, in the muscle, the MUFA content was higher in the TG (D4) and PL fractions (D2) in fish maintained at the lower temperature compared with the higher one within the same diet (Table 5 and Table 6). In the eyes, the MUFA content in the TG fraction of animals from D2-HT was higher than that in D2-LT (Table 9). Fish from groups D1-HT and D4-HT had higher levels of MUFA in the PL composition of the eyes compared to those from the D1-LT and D4-LT groups, respectively (Table 10). In the PL composition of the brain, a higher MUFA content was observed in fish fed D1-HT compared to those fed D1-LT (Table 12), while no differences were observed in the TG fraction (Table 11).

The polyunsaturated FA content did not change in the PL fractions of the muscle, liver, and brain (Table 6, Table 8, and Table 12), and was higher in the eyes of fish maintained at the higher temperature compared to the lower in treatments D3 and D4 (Table 12). In the TG fraction, fish at the lower temperature increased the PUFA content in muscles (Table 5) and liver (Table 7) when fed D2. Additionally, within the higher temperature, the liver content of PUFA was higher in the TG fraction of treatments D3 and D4 compared to D1 (Table 7). A lower content of PUFA was observed in the TG fraction of the brain when fish were maintained at the higher temperature compared to the lower temperature, when fed with the lower DHA level (D1) and compared with the other treatments (D2, D3, and D4), within the higher temperature (Table 11).

The gradual increase in the dietary DHA/EPA ratio resulted in a higher deposition of DHA in the TG of muscle. At the lower temperature, fish fed with the D3 and D4 diets presented a higher DHA level in the muscles (Table 5) and liver (Table 7) compared to the those fed with D1. At the higher temperature, the DHA content was higher in the fish fed with D4 compared to D1 in muscle (Table 5) and compared to D1, D2, and D3 in liver (Table 7). Different DHA/EPA ratios in the experimental diets did not affect DHA levels in the PL of the muscle, eyes, and brain, but increased the DHA in the liver PL fraction in fish fed with D4 compared to D1, D2, and D3 at both temperatures (Table 8). In the TG fraction of the eyes, fish from D4-HT showed a higher DHA content compared to those from D1-HT and D2-HT (Table 9). In the brain, the TG fraction of fish fed with D1 was lower than all other treatments when reared at the higher temperature and within the same diet treatment, compared with fish maintained at the lower temperature (Table 11).

Similar to DHA, the EPA deposition in both lipid classes of muscle tissue reflected the different dietary DHA/EPA ratio. Fish from the D4 group presented a lower EPA deposition in both lipid fractions and temperatures, but the EPA deposition of fish from the D4-HT group was not different from that of D3-HT group in both lipid classes (Table 5 and Table 6). In the liver, the EPA deposition was affected by the different dietary DHA/EPA ratio. Fish from the D4-HT group had the lower EPA levels in TG compared with the other experimental groups at both temperatures (Table 7). Similarly, a gradual EPA decrease in the liver PL was observed, in fish at both temperatures, as the DHA/EPA dietary ratio increased (Table 8). 

The EPA content in the TG of the eyes was not affected by the different dietary DHA/EPA ratio and the different temperatures (Table 9), and the same was observed in the eye PL of fish at LT (Table 10). However, in fish maintained at a high temperature, D3 groups presented a higher EPA content in the PL compared to D1 and D2 (Table 10). In the TG fraction of the brain, the EPA content of D1-HT fish was lower than fish from the D2-HT and D3-HT groups and from D1-LT (Table 11). In the PL, the content of EPA in the brain was higher in fish from the D4-HT group than in D4-LT (Table 12).

### 3.3. Tissue/Diet (T/D) Fatty Acid Ratio

**Figure 1:**
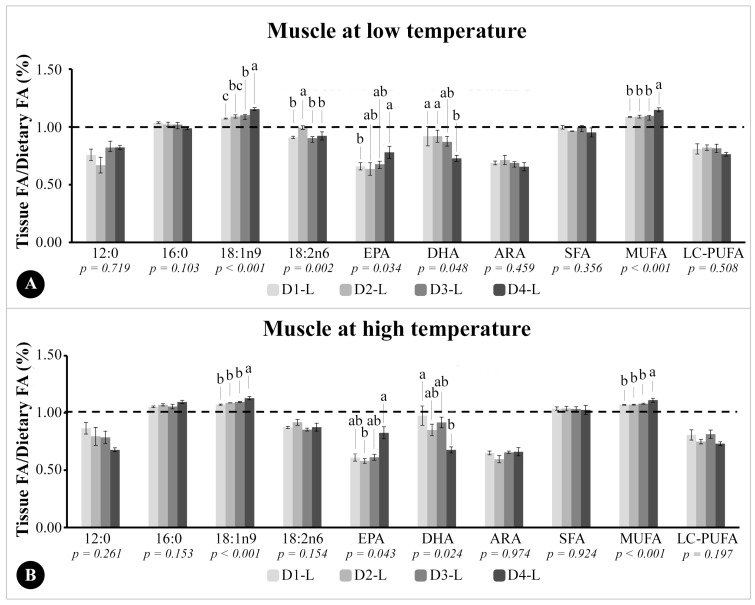
Ratio between the percentage of total fatty acids in the muscle tissue of *Epinephelus marginatus* and the percentage of the same fatty acid in the experimental diet provided during the experimental period. (**A**) D1-LT: Diet 1 (1.00% EPA and 0.50% DHA) at low temperature; D2-LT: Diet 2 (0.75% EPA and 0.75% DHA) at low temperature; D3-LT: Diet 3 (0.50% EPA and 1.00% DHA) at low temperature; D4-LT: Diet 4 (0.00% EPA and 1.50% DHA) at low temperature. (**B**) D1-HT: Diet 1 (1.00% EPA and 0.50% DHA) at high temperature; D2-HT: Diet 2 (0.75% EPA and 0.75% DHA) at high temperature; D3-HT: Diet 3 (0.50% EPA and 1.00% DHA) at high temperature; D4-HT: Diet 4 (0.00% EPA and 1.50% DHA) at high temperature. The dotted line indicates a ratio of 1 to indicate accumulation (greater or less than 1.0) of FA in muscle in relation to the same FA in the diets. ARA: arachidonic acid; DHA: docosahexaenoic acid; EPA: eicosapentaenoic acid; FA: fatty acid; LC-PUFA: long-chain polyunsaturated fatty acids; MUFA: monounsaturated FA; SFA: saturated FA. ^a,b,c^ Different letters indicate statistical differences between the experimental diets, performed using two-way ANOVA; Tukey test (*p* < 0.05).

**Figure 2:**
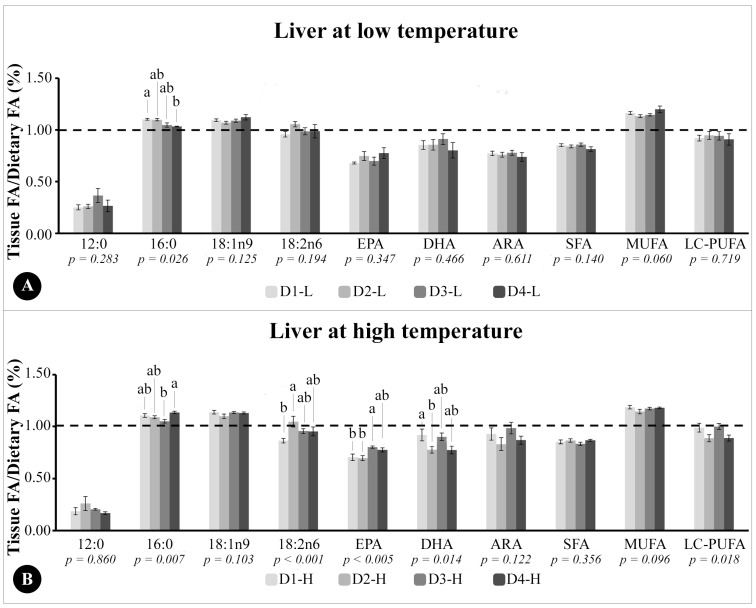
Ratio between the percentage of total fatty acids in the liver tissue of Epinephelus marginatus and the percentage of the same fatty acid in the experimental diet provided during the experimental period. (**A**) D1-LT: Diet 1 (1.00% EPA and 0.50% DHA) at low temperature; D2-LT: Diet 2 (0.75% EPA and 0.75% DHA) at low temperature; D3-LT: Diet 3 (0.50% EPA and 1.00% DHA) at low temperature; D4-LT: Diet 4 (0.00% EPA and 1.50% DHA) at low temperature. (**B**) D1-HT: Diet 1 (1.00% EPA and 0.50% DHA) at high temperature; D2-HT: Diet 2 (0.75% EPA and 0.75% DHA) at high temperature; D3-HT: Diet 3 (0.50% EPA and 1.00% DHA) at high temperature; D4-HT: Diet 4 (0.00% EPA and 1.50% DHA) at high temperature. The dotted line indicates a ratio of 1 to indicate accumulation (greater or less than 1.0) of FA in liver in relation to the same FA in the diets. ARA: arachidonic acid; DHA: docosahexaenoic acid; EPA: eicosapentaenoic acid; FA: fatty acid; LC-PUFA: long-chain polyunsaturated fatty acids; MUFA: monounsaturated FA; SFA: saturated FA. ^ab^ Different letters indicate statistical differences between the experimental diets, performed using two-way ANOVA; Tukey test (*p* < 0.05).

The ratio between the FA in tissue and diets (T/D (%)) of fish muscle at low and high temperatures were presented in Figure 1A,B, respectively. The liver T/D ratios of fish at low and high temperatures were presented in Figure 2A,B, respectively. 

In muscles of fish at a low temperature (Figure 1A), we observed, for all treatments, T/D ratios below 1.0 for 12:0, SFA, ARA, and LC-PUFA, and equal 1.0 for 16:0, and no significant differences were observed when comparing fish fed different experimental diets. The T/D ratio of 18:1n-9 and MUFA were higher than 1.0 in all fish from all groups at the lower temperature. The T/D ratio of 18:1n9 and MUFA was higher in fish from D4-LT compared with all groups at the same temperature. The T/D ratio of DHA in fish muscle at a low temperature was lower than 1.0 in all treatments, and decreased as the DHA/EPA ratio in the experimental diets increased. Fish from the D1-LT and D2-LT groups had higher DHA T/D ratios than fish from the D4-LT group. The EPA T/D ratio in muscle was below 1.0 for all nutritional treatments and higher in the D4-LT group than D1-LT. The T/D ratio of 18:2n6 in muscle was also below 1.0 in all groups at the lower temperature, and higher in D2-LT than the other groups (Figure 1A).

In the muscle of fish maintained at a high temperature (Figure 1B), the T/D ratios of 12:0, 18:2n6, ARA, and LC-PUFA were below 1.0 and did not differ among the different groups. The T/D ratio of 16:0 and SFA were higher than 1.0 and did not differ among the experimental groups. The T/D ratio of 18:1n-9 and MUFA in the muscle of fish at a high temperature also followed the same pattern observed in fish at a low temperature, higher than 1.0, with D4-HT values higher than the other groups. Both EPA and DHA presented T/D ratios below 1.0. The DHA T/D ratio was lower in the muscle of fish from the D4-HT group compared to D1-HT, while the EPA T/D ratio was higher in fish from D4-HT compared to D2-HT (Figure 1B).

In the liver of the fish maintained at a low temperature (Figure 2A), the T/D ratio of 12:0, EPA, DHA, ARA, SFA, and LC-PUFA did not change according to the treatment and were below 1.0. The T/D ratio of 18:1n9 and MUFA were higher than 1.0 in all groups and did not change, while the T/D ratio of 18:2n6 was near 1.0 for most groups and did not change among the different treatments. The only difference among groups observed in the T/D ratio of the liver in fish maintained at a low temperature was observed in 16:0, with fish from D1-LT presenting higher values than D4-LT (Figure 2A).

In the liver of fish at a high temperature (Figure 2B), the T/D ratio of 12:0, ARA, SFA, and LC-PUFA did not change according to the treatment and were below 1.0. The T/D ratio of 18:1n9 and MUFA were higher than 1.0 in all groups and did not change. The T/D ratio of EPA was below 1.0 in all groups, and higher in the liver of D3-HT fish than that of D1-HT and D2-HT, while the T/D ratio of DHA, also below 1.0, was higher in the liver of D1-HT fish than D2-HT. The T/D ratio of 16:0 was above 1.0 in the liver of all groups at a high temperature and was higher in D4-HT than in D3-HT (Figure 2B).

### 3.4. Liver Histology Analysis

**Figure 3:**
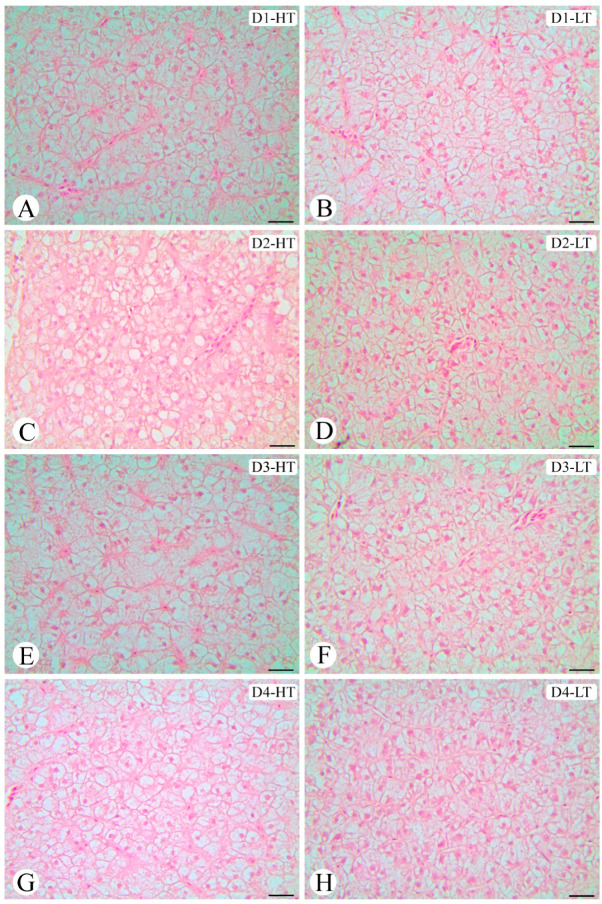
Morphologies of the hepatocytes of *Epinephelus marginatus* fed different experimental diets in different temperatures. (**A**) D1-HT: Diet 1 (1.00% EPA and 0.50% DHA) at high temperature; (**B**) D1-LT: Diet 1 (1.00% EPA and 0.50% DHA) at low temperature; (**C**) D2-HT: Diet 2 (0.75% EPA and 0.75% DHA) at high temperature; (**D**) D2-LT: Diet 2 (0.75% EPA and 0.75% DHA) at low temperature; (**E**) D3-HT: Diet 3 (0.50% EPA and 1.00% DHA) at high temperature; (**F**) D3-LT Diet 3 (0.50% EPA and 1.00% DHA) at low temperature; (**G**) D4-HT: Diet 4 (0.00% EPA and 1.50% DHA) at high temperature; (**H**) D4-LT: Diet 4 (0.00% EPA and 1.50% DHA) at low temperature. (*n* = 3 fish/tank; *n* = 45 cells/fish measured; 135 cells per experimental group; with 100 μm distance between each section; totaling 300 μm distance from the first section to the last part). DHA: docosahexaenoic acid; EPA: eicosapentaenoic acid. Haematoxylin–eosin staining. Bars: 25 μm.

**Figure 4:**
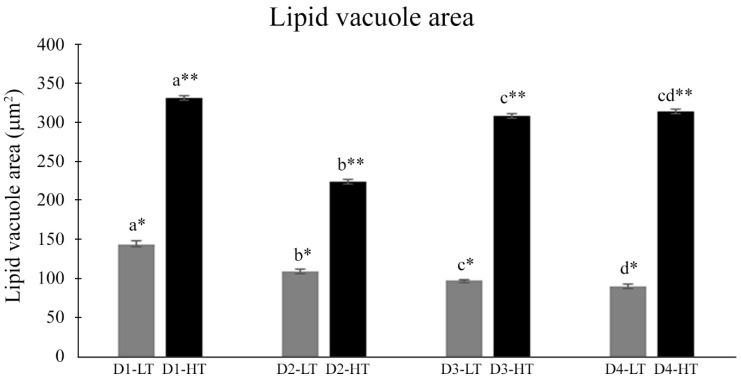
Hepatocytes average size (μm^2^) of *Epinephelus marginatus* fed with different experimental diets in different temperatures. D1-HT: Diet 1 (1.00% EPA and 0.50% DHA) at high temperature; D2-HT: Diet 2 (0.75% EPA and 0.75% DHA) at high temperature; D3-HT: Diet 3 (0.50% EPA and 1.00% DHA) at high temperature; D4-HT: Diet 4 (0.00% EPA and 1.50% DHA) at high temperature; D1-LT: Diet 1 (1.00% EPA and 0.50% DHA) at low temperature; D2-LT: Diet 2 (0.75% EPA and 0.75% DHA) at low temperature; D3-LT: Diet 3 (0.50% EPA and 1.00% DHA) at low temperature; D4-LT: Diet 4 (0.00% EPA and 1.50% DHA) at low temperature. DHA: docosahexaenoic acid; EPA: eicosapentaenoic acid. ^a,b,c,d^ Different letters indicate statistical differences between the experimental diets at the same temperature. *, ** Different symbols indicate statistical differences between the same experimental diets but in different temperature (low or high temperature). These analyses were performed using two-way ANOVA; Tukey test (*p* < 0.05).

The hepatic morphology and lipid vacuole area of the liver cells of *E. marginatus* juveniles fed different experimental diets and kept at different temperatures are presented in Figure 3 and Figure 4, respectively. The interaction of dietary treatments and temperature changed the hepatocyte morphology (Figure 3). Fish at the higher temperature had larger lipid vacuole areas than fish at the lower temperature (*p* < 0.001), regardless of the experimental diet (Figure 4). Fish from D1-HT showed larger vacuole areas than other groups (D2-HT, D3-HT, and D4-HT) (*p* < 0.001), and fish from the D2-HT diet presented smaller vacuole areas compared to D3-HT (*p* < 0.001). Fish maintained at the lower temperature presented a similar distribution pattern as those maintained at a high temperature, with D1-LT showing larger lipid vacuole areas, and D4-LT smaller (*p* < 0.001) (Figure 4).

## 4. Discussion

The results obtained in this study showed that different dietary DHA/EPA levels did not affect the growth performance of *E. marginatus* at the lower temperature but affect the growth performance at the higher temperature, with a better DGR and WG in fish fed with the higher DHA level. This result suggests that dusky grouper has a lower nutritional requirement of DHA when kept at 23.17 ± 0.12 °C, possibly not being necessary to include this FA in greater levels in the colder seasons, such as autumn and winter. However, at 28.63 ± 0.06 °C, the species showed better growth performance when fed with a higher level of DHA, possibly indicating that, in warmer seasons, such as spring and summer, a greater inclusion of DHA is necessary for the better growth of juveniles. Previous studies with *Salmo salar* [37], *Oncorhynchus mykiss* [38], *R. canadum* [17,39], and *M. saxatilis* [33] also demonstrated different requirements of LC-PUFA in the diet according to rearing conditions, such as temperature. These factors can directly influence species growth, also impacting LC-PUFA levels in tissues, as observed in *Tor putitora*, a tropical fish species that showed higher levels of DHA at a high temperature (32 °C) [40]. Jobling and Bendiksen [23] further describe that, in fish, as ectothermic animals, temperature can define the rates of chemical reactions that occur in these organisms, and, thus, dictate the speed with which physiological processes occur. As a result, several metabolic processes, such as cellular respiration and oxygen consumption, may undergo changes. Therefore, the standard metabolic rate (SMR) can be affected (high temperature = high SMR; low temperature = low SMR), while the maximum metabolic rate (MMR) can vary and stabilize. And when animals are able to maintain their vital activities, much of the energy is directed towards functions such as growth, for example.

It is important to highlight that all diets in this experiment were fish-oil-free and supplemented with VO (coconut oil and olive oil), and this FO substitution did not impact the growth performance and survival of *E. marginatus* juveniles. Studies with different fish species, such as *Argyrosomus regius* [41], *Totoaba mcdonald* [16], *Trachinotus ovatus* [42], *R. canadum* [25], and even species of the genus *Epinephelus* [18,43,44,45,46], also addressed the importance of replacing FO with VO, suggesting that different species have specific physiological requirements for DHA and EPA. Therefore, although the inclusion of FO is a traditional practice in marine fish nutrition, it is possible to replace it with VO since the diets were adequately supplemented with LC-PUFA n3, such as DHA and EPA [14], as performed in this study. Moreover, FA, such as DHA and EPA are essential for marine fish, especially in captivity, as they depend on food to obtain them and cannot synthesize them again [15]. These LC-PUFA can be used to obtain energy through β-oxidation but are mainly present in biological membranes. β-oxidation is a four-step process that results in acetyl-CoA or ketone bodies. These molecules are made available to the organism for various processes and tissues, which can use them in the most optimized way possible, such as in the process of growth and reproduction. Nervous tissue, for example, can use ketone bodies in the absence of glucose to obtain energy. The energy obtained aids growth as the organism has sufficient energy sources in the system to distribute as needed. Furthermore, as previously stated, DHA and EPA are FAs present in biological membranes that configure the fluidity and proper functioning of the lipid bilayer, as mentioned in the literature referenced throughout the manuscript. Furthermore, the fluidity in cell membranes gives them the ability to fuse, and among the processes that we can mention are endo- and exocytosis, the fertilization of the egg by a sperm, cell division, and other processes [13]. Other factors are important for the occurrence of physiological processes, such as temperature, which can be an ally, favoring and facilitating the progress of processes, or can trigger imbalances in these processes, making their occurrence difficult. As the environmental temperature is modified, one of the only cellular components that can be remodeled is the plasma membrane, through the remodeling/restructuring of the lipid composition, called homeoviscosal adaptation [12]. The LC-PUFA EPA, DHA, and ARA help maintain membrane fluidity at low temperatures. Due to their low melting point, they occupy more space in the lipid bilayer, increasing the fluidity and stability of the plasma membrane [12,13]. When the temperature is high, the membrane becomes even more fluid. Saturated fats, such as those provided by coconut oil, tend to become more rigid at temperatures of 25°C, while unsaturated fats, such as those provided by olive oil, tend to remain fluid; the same happens with the LC-PUFA DHA and EPA, which are unsaturated [14]. Therefore, fish fed diets supplemented with LC-PUFAs may present greater thermal tolerance, maximizing growth and survival, since the constitution and functioning of cell membranes can directly affect the maintenance of physiological homeostasis and the immune system of fish [12,13,14].

In the TG fraction of muscle, DHA and EPA levels reflected dietary levels; however, the T/D ratio indicated that DHA and EPA were not proportionally retained in muscular tissue, especially in fish fed the highest levels of DHA (D4). Similarly, in *T. ovatus* [42] and *E. cocoides* [18], the DHA/EPA ratio above 1.00 did not result in a greater deposition of DHA in the muscle. Thus, the results of this study suggest that, for better levels of DHA deposition in *E. marginatus* fillets, the DHA/EPA ratio should be close to 1.00 and less than 3.00. Moreover, the physiological requirement for LC-PUFA is different from the necessity to maintain high levels of LC-PUFA in tissues [47]. Thus, the dietary inclusion of DHA and EPA should be assessed in commercially relevant marine species aiming to make a better use of marine resources. Moreover, in marine fish, the oxidation of LC-PUFA is stimulated when the dietary levels of these substrates are in excess [48].

In the liver, both fractions reflected the DHA present in the diet and, similar to muscle, PL had higher levels of LC-PUFA than TG, especially DHA. This result is consonant with the studies conducted so far that suggest that DHA in the polar lipids of fish tissues is highly conserved in the liver and muscle, among other tissues [49,50,51]. Although the different dietary DHA/EPA ratios did not affect the sum of LC-PUFA in the liver, when fish were fed 0.5 of EPA and 1.0 of DHA (D3), the levels of DHA deposition were not different from those fish that received 1.5 of DHA (D4). Possibly, the balance in the proportion of these FAs in the metabolism is more important than the amount itself. Thus, increasing dietary DHA levels and depriving fish of EPA affect the FA profile in TG and PL. Studies with *T. ovatus* have shown that DHA is important in the immune system and that lysozyme activity increases as dietary DHA increases. However, lysozyme activity decreased when dietary DHA levels were much higher than those of EPA (2.2) [42]. Similar results were observed in large yellow croaker, *Larmichthys crocea*; when dietary LC-PUFA n-3 increased from 1.37% to 2.25%, lysozyme activity decreased [52]. DHA is important in numerous metabolic and physiological processes in animals, including marine fish, so receiving a proper content of DHA in the diet is imperative [15,19]. However, excessive levels of dietary DHA could reduce immune parameters such as lysozyme activity or even have adverse effects in fish tissue such as hepatic lesions, as described in European sea bass, *Dicentrarchus labrax* [53].

EPA levels in both hepatic fractions, TG and PL, reflected the diet, and decreased as the dietary DHA/EPA ratio increased. The reduction of EPA, especially in the PL composition, could be prejudicial to the immunological response of *E. marginatus* juveniles, as EPA is a mediator of inflammatory processes, and, through phospholipase, A2 action is cleaved from PL to synthesize eicosanoids of the anti-inflammatory series [54,55]. In fish, the immunological response mediated by eicosanoids is partially related to the EPA and ARA levels, which are important in the regulation of the inflammatory response. The n-3/n-6 ratio in the feed is an index to consider for feed formulation, as imbalances can trigger adverse physiological consequences [56,57,58].

The diet profile of the DHA/EPA ratio was less reflected in the organs of the nervous system analyzed in this study. In the eyes, the DHA content of the PL fraction was not affected by the diet, but when fish were fed with the highest DHA inclusion and maintained at the higher temperature (D4-HT), a remodeling of FA was observed, increasing MUFA (especially 18:1n-9) and PUFA (not a specific FA) levels and causing a decrease in SFA (especially 16:0). Environmental factors such as water temperature affect the metabolic rate of fish [59], and, in response to fluctuations in water temperature and the metabolic rate, fish can restructure the phospholipid and FA composition of cell membranes toward increasing fluidity [24]. In this context, MUFA and PUFA play an important role in membrane composition, as the presence of unsaturation in the membrane creates “bends” in the lipid layers that ultimately support membrane fluidity [60]. Our results suggest that juvenile of *E. marginatus*, due to higher metabolic demand at the higher temperature, deposited higher levels of MUFA and PUFA in the PL fraction of the eyes, since the increase in the temperature increases the metabolic rate of fish, and, this tissue, belonging to the nervous system, is very energy-consuming. However, this profile was not observed in the brain. Consonant with this pattern, in the TG fraction of the eyes, the levels of 18:0 decreased at a high temperature. The TG are molecules used by animals, including teleosts, as energy stores [13], and SFA are preferentially oxidized due to their fast energy delivery [61]. Thus, fish maintained at a high temperature possibly increase the metabolic rate and SFA oxidation to balance the energy demand.

In general, LC-PUFA levels were preserved in these tissues regardless of the temperature or the levels at which these FAs were included in the diet. This pattern reflects the importance of DHA and EPA in nervous tissues such as the brain and eyes of marine teleosts. The results demonstrated that, in *E. marginatus* juveniles, DHA is more preserved than EPA, especially in the PL of these tissues, in which there are no differences in the levels of DHA content. This profile was expected, and the fact that the FA profile of PL is more conserved, especially in nervous tissues, regardless of the diet, has already been observed in fish [62]. In the present study, it was also observed that, even when fed higher (D1) or equal (D2) levels of EPA than DHA, fish retained more DHA, suggesting that EPA is more oxidized, as was also observed in *R. canadum* [63]. Beyond metabolic alterations, our study also revealed morphological alterations in the liver, both related to temperature and the dietary DHA/EPA ratio. The liver is a key organ in fish metabolism [13] and energy modulation is vital for fish at a low temperature [64]. Cold stress can lead to considerable energy expenditure [65] and this probably explains the reduced lipid deposition pattern and hepatic lipid droplet area of fish kept at a low temperature compared to fish kept at a high temperature. Moreover, the reduction in lipid droplet area was related to an increased dietary DHA/EPA ratio, a result that corroborates the FA profile, which showed a decrease in EPA content in liver levels of fish kept at a low temperature as the dietary DHA/EPA increased. On the other hand, the higher deposition of lipid droplets in fish kept at a high temperature could be related to the increase of lipid deposition in the liver of fish kept at this temperature; however, when maintained at the higher temperature, there is no difference in the lipid vacuole area of fish fed D3 and D4, suggesting that there is a limit for DHA deposition from dietary intake.

Some studies have been performed aiming to, through nutritional manipulation, increase DHA and EPA retention in the fillets of commercial marine fish species [25,66,67] due to consumer pressure for a healthier product [47], as these FAs promote benefits in human health [68,69]. Considering that: (1) aquaculture is the unique way to offer dusky grouper fillet to the market, considering the conservation state of this species [2]; (2) the production of aquafeeds is one of the most expensive points in the productive cycle [70]; and (3) alternative ingredients to FO such as algal oils are still difficult to scale and have a costly production [47], studies that access the dietary ratio of DHA/EPA in marine fish nutrition are important in order to better understand the physiology of these species and further improve fillet quality.

## 5. Conclusions

Our study showed that VO oils, such as coconut and olive oil, can replace FO in dusky grouper juvenile diets without impairing growth performance, as long as the diet is properly supplemented with DHA and EPA. The different dietary DHA/EPA ratio triggered different patterns of FA deposition in the lipid fractions of the organs, with the FA profile in TG reflecting the dietary FA profile, while the FA profile of the PL fraction was less influenced by the diet. The increase in the DHA/EPA dietary ratio did not result in a greater deposition of DHA in muscular tissue, evidencing that, in the diet formulation, the balance of these LC-PUFA is as important as the quantity.

The FA profile in the brain and eyes was slightly modified by the dietary DHA/EPA ratio; however, it was possible to observe a sign of homeoviscous adaptation in the eyes, remodeling the membrane with MUFA and PUFA instead of SFA, suggesting a regulation of the membrane fluidity, and increasing oxidation to cope with the metabolic demand of a high temperature. Finally, the temperature influenced the hepatocyte morphology, reducing the area of the lipid vacuoles in fish kept at a low temperature (23.17 ± 0.12 °C) and increasing the area in fish kept at a high temperature (28.63 ± 0.06 °C), an outcome that is important for generating information about the basic aspects of dusky grouper aquaculture, mainly for Brazilian aquaculture. These results evidence the importance of both temperature and a balanced DHA/EPA ratio in the diet of marine fish, and support the fact that the dietary inclusion of DHA and EPA should be assessed in commercially relevant marine species aiming to make a better use of marine resources.

## Figures and Tables

**Table 1:** Formulation and proximate composition of experimental diets (g kg^−1^) containing different proportion of DHA (22:6n-3) and EPA (20:5n-3).

Ingredients	Dietary DHA/EPA Levels (g kg^−1^)			
	D1	D2	D3	D4
Defatted poultry meal ^a^	40.0	40.0	40.0	40.0
Defatted fish meal ^b^	20.0	20.0	20.0	20.0
Squid meal ^c^	10.0	10.0	10.0	10.0
Wheat flour	14.0	14.0	14.0	14.0
Blood meal	5.5	5.5	5.5	5.5
Soy lecithin	1.0	1.0	1.0	1.0
Premix min. and vit. ^d^	3.0	3.0	3.0	3.0
Stay C	0.5	0.5	0.5	0.5
Taurine	0.8	0.8	0.8	0.8
Sodium benzoate	0.2	0.2	0.2	0.2
BHT	0.01	0.01	0.01	0.01
Coconut oil ^e^	2.0	2.0	2.0	2.0
Olive oil ^f^	1.2	1.2	1.2	1.2
EPA oil 45% ^g^	1.00	0.75	0.50	0.00
ARA oil 40% ^h^	0.30	0.30	0.30	0.30
DHA oil 50% ^i^	0.50	0.75	1.00	1.50
*Proximate composition* (g kg^−1^)				
Crude lipid	108.20	111.20	103.60	99.30
Crude protein	523.10	527.10	514.40	510.90
Dry matter	76.20	67.30	77.40	96.80
Ash	136.60	140.20	137.40	134.60
NFE	155.90	154.20	167.20	158.40

DHA: docosahexaenoic acid (22:6n-3); EPA: eicosapentaenoic acid (20:5n-3); ARA: arachidonic acid (20:4n-6); NFE (g kg^−1^) including fiber = 1000 − (crude protein + crude lipid + Ash); ^a, b, c, d, e, f, g, h, i^ Nutricon Ltd.a-Me, São Paulo, Brazil; ^a^ Defatted poultry meal (defatted by 3x hexane extraction)—poultry meal of South American origin; ^b^ Defatted fish meal (defatted by 3x hexane extraction)—fish meal of South American origin; ^c^ Squid meal of South American origin; ^d^ Vitamin and mineral premix (UI kg^−1^ or g kg^−1^ of premix): vitamin A, 2.5 MIU; vitamin D3, 0.25 MIU; vitamin E, 16.7 g; vitamin K3, 1.7 g; vitamin B1, 2.5 g; vitamin B2, 4.2 g; vitamin B3, 25 g; vitamin B5, 8.3 g; vitamin B6, 2.0 g; vitamin B9, 0.8; vitamin B12, 0.005 g; biotin, 0.17 g; vitamin C, 75 g; choline, 166.7 g; inositol, 58.3 g; ethoxyquin, 20.8 g; copper, 2.5 g; ferrous iron, 10.0 g; magnesium, 16.6 g; manganese, 15.0 g; zinc, 25.0 g; ^e^ Copra Indústria Alimentícia Ltd.a., Alagoas, Brazil; ^f^ Victor Guedes, Ind. Com. S.A., Abrantes, Portugal; ^g^ EPA concentrated fish oil (>45% EPA), Phosphotech Laboratories, ZAC de la Lorie, France; ^h^ ARA concentrated oil (>40% ARA), Jangsu Tiankai Biotechnology Co., Ltd., Nanjing, China. ^i^ Incromega™ DHA 500 TG (>50% DHA), CRODA™, Snaith, East Yorkshire, UK.

**Table 2:** Fatty acid profile of the experimental diets and oils (% of total fatty acids).

Fatty Acids (%)	Diets (%)				Oils (%)		
	D1	D2	D3	D4	ARA	EPA	DHA
12:0	6.49	6.88	6.53	6.45	nd.	nd.	nd.
14:0	4.04	4.15	4.10	4.13	2.29	0.49	0.78
16:0	17.79	17.63	17.97	17.87	8.96	20.44	18.96
16:1n-7	3.11	3.15	3.23	3.22	0.11	0.36	0.28
18:0	6.38	6.38	6.40	6.27	9.70	0.38	0.75
18:1n-9	33.58	33.43	32.87	32.37	28.02	1.35	0.34
18:1n-7	2.36	2.40	2.35	2.25	1.46	0.79	nd.
18:2n-6	12.56	11.72	12.53	12.43	nd.	nd.	nd.
18:3n-3	1.38	1.28	1.34	1.34	nd.	nd.	0.26
18:4n-3	0.35	0.34	0.31	0.27	nd.	nd.	nd.
20:1n-9	1.42	1.40	1.31	1.24	0.18	nd.	0.19
20:2n-6	0.46	0.46	0.44	0.45	nd.	nd.	nd.
20:4n-6	1.69	1.75	1.66	1.70	44.95	nd.	nd.
20:5n-3	4.13	3.73	3.10	2.04	nd.	53.15	11.53
22:5n-3	0.59	0.67	0.71	0.86	nd.	nd.	nd.
22:6n-3	3.69	4.64	5.15	7.11	nd.	12.59	60.27
∑SFA	34.69	35.05	35.00	34.72	21.82	21.31	20.70
∑MUFA	40.47	40.38	39.76	39.08	29.79	2.50	0.81
∑PUFA	24.84	24.57	25.25	26.20	44.95	65.74	72.06
∑LC-PUFA	10.55	11.24	11.06	12.16	44.95	65.74	72.06
∑n-3 PUFA	10.14	10.65	10.61	11.62	nd.	65.74	72.06
∑n-6 PUFA	14.70	13.92	14.63	14.58	44.95	nd.	nd.
DHA/EPA	0.89	1.24	1.66	3.49			

∑SFA, ∑MUFA, ∑PUFA, ∑LC-PUFA, ∑n-3 PUFA, and ∑n-6 PUFA are the sum of saturated (SFA), monosaturated (MUFA), polyunsaturated (PUFA), long-chain polyunsaturated fatty acids (LC-PUFA), polyunsaturated n3 (n-3 PUFA), and polyunsaturated n6 (n-6 PUFA), respectively. DHA/EPA is the ratio between the sum of total DHA (docosahexaenoic acid, 22:6n-3) and EPA (eicosapentaenoic acid, 20:5n-3). nd. = not detected.

**Table 3:** Effect of dietary fatty acid (FA) composition and temperature (LT—lower temperature; and HT—higher temperature) on growth performance, survival, and body parameters of *Epinephelus marginatus* juveniles.

Index	Dietary FA Source/Temperature (Lower and Higher)								*p*-Values		
	D1-LT	D1-HT	D2-LT	D2-HT	D3-LT	D3-HT	D4-LT	D4-HT	T	D	D × T
Initial weight (g)	37.42 ± 2.10	39.90 ± 2.13	38.52 ± 1.86	37.21 ± 1.78	37.71 ± 1.93	41.52 ± 1.98	38.76 ± 2.12	39.75 ± 1.98	0.289	0.828	0.610
Final weight (g)	80.74 ± 4.42	91.69 ± 5.60 ^ab^	81.11 ± 3.72	78.33 ± 3.95 ^a^	82.04 ± 4.36	87.59 ± 4.77 ^ab^	82.50 ± 4.95 *	100.26 ± 5.02 ^b^**	0.017	0.097	0.158
WG (g) ^1^	43.32 ± 4.62	51.78 ± 2.62	42.60 ± 0.74	41.12 ± 4.36	44.33 ± 3.25	46.07 ± 6.93	43.60 ± 4.88 *	60.51 ± 2.06 **	0.042	0.129	0.160
DGR (g/day) ^2^	0.72 ± 0.08	0.86 ± 0.04	0.71 ± 0.01	0.69 ± 0.07	0.74 ± 0.05	0.77 ± 0.12	0.73 ± 0.08 *	1.01 ± 0.03 **	0.042	0.129	0.160
SGR (%d) ^3^	1.28 ± 0.09	1.39 ± 0.04	1.24 ± 0.02	1.24 ± 0.01	1.29 ± 0.05	1.23 ± 0.12	1.25 ± 0.12	1.54 ± 0.02	0.156	0.222	0.174
HSI (%) ^4^	1.25 ± 0.08	1.50 ± 0.14	1.15 ± 0.08	1.29 ± 0.11	1.09 ± 0.08	1.35 ± 0.15	1.06 ± 0.09	1.23 ± 0.10	0.008	0.194	0.933
SR (%) ^5^	100 ± 0.00	100 ± 0.00	100 ± 0.00	100 ± 0.00	100 ± 0.00	100 ± 0.00	100 ± 0.00	100 ± 0.00	1.000	1.000	1.000

Values represent means ± standard error of the mean (to calculate initial and final weights, weight gained, daily gain rate, specific growth rate, and survival rate, all fish were used (*n* = 9/tank); to calculate HSI index, 3 fish/tank were used. ^a,b^ Different letters indicate statistical differences between experimental diets at the same temperature, using Tukey’s test (*p* < 0.05). *, ** Symbol indicates statistical differences within the same diet at different temperatures by Tukey’s test (*p* < 0.05). T represents *p*-value of temperature; D represents *p*-value of diet; and DxT represents *p*-value of the interaction diet versus temperature. ^1^ Weight gained (WG, g) = W_f_ − W_i_. ^2^ Daily gain rate (DGR, g/day) = (W_f_ − W_i_)/t. ^3^ Specific growth rate (SGR, %d) = (LnW_f_ − LnW_i_) × 100/t. ^4^ Hepatosomatic index (HSI, %) = 100 × (liver weight/W_f_). ^5^ Survival rate (SR, %) = N_f_ × 100/N_i_. Where W_f_ and W_i_ were final and initial fish weight, respectively; N_f_ and N_i_ were final and initial number of fish, respectively; and t was duration of experiment in days.

**Table 4:** Total lipids (mg g^−1^ of wet weight) of *Epinephelus marginatus* juvenile fed different experimental diets in different temperatures (LT—lower temperature; HT—higher temperature).

Tissues	Dietary FA Source/Temperature (Lower or Higher)								*p*-Values		
	D1-LT	D1-HT	D2-LT	D2-HT	D3-LT	D3-HT	D4-LT	D4-HT	T	D	D × T
Muscle	17.85 ± 1.86	15.30 ± 1.55	16.47 ± 1.63	20.04 ± 1.76	15.51 ± 2.36	13.41 ± 1.08	23.96 ± 2.79 *	14.90 ± 0.90 **	0.023	0.172	0.016
Liver	93.27 ± 3.35 ^a^*	116.17 ± 5.69 **	81.24 ± 3.31 ^b^*	93.84 ± 2.65 **	90.99 ± 7.02 ^a^*	111.62 ± 4.31 **	86.19 ± 3.58 ^a^*	113.70 ± 3.74 **	<0.001	0.001	0.353
Eyes	41.96 ± 1.88	35.25 ± 2.16	41.71 ± 3.07	42.61 ± 3.63	35.93 ± 5.10	28.82 ± 1.71	41.76 ± 3.78	38.30 ± 3.68	0.091	0.428	0.602
Brain	85.07 ± 2.05 *	70.65 ± 4.49 **	83.76 ± 3.22	78.80 ± 4.69	83.03 ± 4.04 *	67.92 ± 2.93 **	84.13 ± 3.61	72.76 ± 2.93	<0.001	0.873	0.245

Values represent means ± standard error of the mean (*n* = 3 animals per tank; *n* = 3 tanks by treatments). ^a,b^ Different letters indicate statistical differences between experimental diets by Tukey’s test (*p* < 0.05). *, ** Symbol indicates statistical differences within the same diet at different temperatures by Tukey’s test (*p* < 0.05). T represents *p*-value of temperature; D represents *p*-value of diet; and DxT represents *p*-value of diet versus temperature.

**Table 5:** Fatty acid composition of triacylglycerol in the muscle of *Epinephelus marginatus* juveniles fed different experimental diets in different temperatures (LT—lower temperature; HT—higher temperature).

Fatty Acid	Dietary FA Source/Temperature (Lower or Higher)								*p*-Values		
	D1-LT	D1-HT	D2-LT	D2-HT	D3-LT	D3-HT	D4-LT	D4-H	T	D	D × T
12:0	4.92 ± 0.23	5.55 ± 0.35	4.52 ± 0.74	5.51 ± 0.41	5.37 ± 0.31	5.13 ± 0.59	5.34 ± 0.27	4.37 ± 0.34	0.722	0.731	0.089
14:0	5.06 ± 0.12	5.36 ± 0.12	4.89 ± 0.29	5.63 ± 0.23	5.18 ± 0.14	5.60 ± 0.29	4.92 ± 0.69	5.19 ± 0.17	0.054	0.732	0.867
16:0	18.43 ± 0.17	18.71 ± 0.20	18.11 ± 0.24 *	18.85 ± 0.19 **	18.32 ± 0.27	18.95 ± 0.32	17.73 ± 0.21 *	19.58 ± 0.30 **	<0.001	0.899	0.013
16:1	3.56 ± 0.10	3.34 ± 0.05	3.37 ± 0.07	3.19 ± 0.08	3.38 ± 0.08	3.26 ± 0.09	3.47 ± 0.08	3.34 ± 0.04	0.005	0.056	0.740
18:0	6.19 ± 0.07	6.31 ± 0.09	6.23 ± 0.32	6.40 ± 0.25	5.95 ± 0.22	6.47 ± 0.15	5.44 ± 0.10 *	6.46 ± 0.24 **	0.002	0.267	0.096
18:1n-9	36.03 ± 0.19 ^a^	35.97 ± 0.19	36.46 ± 0.37 ^ab^	36.35 ± 0.12	36.20 ± 0.17 ^a^	36.01 ± 0.21	37.27 ± 0.28 ^b^	36.52 ± 0.24	0.091	<0.001	0.386
18:1n-7	2.96 ± 0.09	2.61 ± 0.05	2.82 ± 0.11	2.43 ± 0.06	2.65 ± 0.08	2.48 ± 0.07	2.70 ± 0.10 *	2.27 ± 0.29 **	0.001	0.177	0.791
18:2n-6	11.43 ± 0.13	10.97 ± 0.16	11.64 ± 0.22 *	10.72 ± 0.27 **	11.32 ± 0.18 *	10.69 ± 0.26 **	11.36 ± 0.27	10.87 ± 0.20	<0.001	0.809	0.701
18:3n-3	1.18 ± 0.03	1.13 ± 0.03	1.21 ± 0.05 *	1.08 ± 0.05 **	1.15 ± 0.03	1.07 ± 0.04	1.14 ± 0.04	1.09 ± 0.03	0.005	0.508	0.682
18:4n-3	0.24 ± 0.03 ^a^	0.19 ± 0.01	0.19 ± 0.02 ^ab^	0.16 ± 0.02	0.18 ± 0.02 ^b^	0.15 ± 0.01	0.14 ± 0.01 ^b^	0.17 ± 0.03	0.304	0.043	0.329
20:1n-9	1.46 ± 0.06	1.35 ± 0.04	1.39 ± 0.06	1.25 ± 0.06	1.30 ± 0.05	1.23 ± 0.05	1.26 ± 0.06	1.27 ± 0.05	0.067	0.064	0.534
20:2n-6	0.46 ± 0.03	0.42 ± 0.01	0.46 ± 0.03	0.39 ± 0.03	0.40 ± 0.02	0.39 ± 0.03	0.46 ± 0.05	0.40 ± 0.02	0.042	0.431	0.786
20:4n-6	1.16 ± 0.03	1.11 ± 0.03	1.24 ± 0.08 *	1.03 ± 0.04 **	1.14 ± 0.03	1.08 ± 0.04	1.10 ± 0.04	1.12 ± 0.03	0.013	0.872	0.056
20:5n-3	2.72 ± 0.07 ^a^	2.54 ± 0.07 ^a^	2.40 ± 0.18 ^ab^	2.14 ± 0.13 ^ab^	2.10 ± 0.06 ^b^	1.91 ± 0.08 ^b^	1.60 ± 0.10 ^c^	1.68 ± 0.22 ^b^	0.150	<0.001	0.573
22:5n-3	0.81 ± 0.03	0.87 ± 0.03	0.87 ± 0.05	0.87 ± 0.03	0.88 ± 0.04	0.90 ± 0.05	0.94 ± 0.04	0.86 ± 0.03	0.935	0.458	0.335
22:6n-3	3.39 ± 0.19 ^a^	3.57 ± 0.34 ^a^	4.20 ± 0.36 ^ab^	3.99 ± 0.33 ^ab^	4.48 ± 0.16 ^b^	4.65 ± 0.39 ^ab^	5.14 ± 0.33 ^b^	4.81 ± 0.17 ^b^	0.812	<0.001	0.737
∑SFA	34.60 ± 0.39	35.93 ± 0.34	33.75 ± 0.72 *	36.39 ± 0.76 **	34.81 ± 0.58	36.16 ± 0.57	33.43 ± 0.82 *	35.59 ± 0.36 **	<0.001	0.380	0.636
∑MUFA	44.00 ± 0.28	43.27 ± 0.17	44.05 ± 0.40	43.22 ± 0.20	43.53 ± 0.27	42.99 ± 0.33	44.70 ± 0.40 *	43.40 ± 0.20 **	<0.001	0.062	0.587
∑PUFA	21.40 ± 0.32	20.80 ± 0.45	22.20 ± 0.71 *	20.39 ± 0.61 **	21.66 ± 0.41	20.85 ± 0.51	21.87 ± 0.51	21.01 ± 0.31	0.004	0.911	0.616
∑LC-PUFA	8.55 ± 0.27	8.50 ± 0.39	9.17 ± 0.53	8.43 ± 0.36	9.01 ± 0.25	8.94 ± 0.43	9.23 ± 0.32	8.88 ± 0.21	0.213	0.420	0.733
∑n-3 PUFA	8.36 ± 0.28	8.30 ± 0.37	8.86 ± 0.46	8.25 ± 0.34	8.80 ± 0.25	8.69 ± 0.41	8.95 ± 0.30	8.61 ± 0.21	0.231	0.484	0.823
∑n-6 PUFA	13.05 ± 0.15	12.50 ± 0.17	13.34 ± 0.32 *	12.14 ± 0.33 **	12.87 ± 0.22	12.16 ± 0.30	12.92 ± 0.32	12.40 ± 0.22	<0.001	0.756	0.547
DHA/EPA	1.25 ± 0.06 ^a^	1.42 ± 0.16 ^a^	1.83 ± 0.27 ^ab^	1.96 ± 0.30 ^ab^	2.13 ± 0.06 ^b^	2.48 ± 0.30 ^bc^	3.34 ± 0.29 ^c^	3.22 ± 0.35 ^c^	0.461	<0.001	0.816

Values represent means ± standard error of the mean (*n* = 3 animals per tank; *n* = 3 tanks by treatments). ^a,b,c^ Different letters indicate statistical differences between experimental diets by Tukey’s test (*p* < 0.05). *, ** Symbol indicates statistical differences within the same diet at different temperatures by Tukey’s test (*p* < 0.05). T represents *p*-value of temperature; D represents *p*-value of diet; and DxT represents *p*-value of diet versus temperature. ∑SFA, ∑MUFA, ∑PUFA, ∑LC-PUFA, ∑n-3 PUFA, and ∑n-6 PUFA are the sum of saturated (SFA), monosaturated (MUFA), polyunsaturated (PUFA), long-chain polyunsaturated fatty acids (LC-PUFA), polyunsaturated n3 (n-3 PUFA), and polyunsaturated n6 (n-6 PUFA), respectively. DHA/EPA is the ratio between the sum of total DHA (docosahexaenoic acid, 22:6n-3) and EPA (eicosapentaenoic acid, 20:5n-3).

**Table 6:** Fatty acid composition of phospholipids in the muscle of *Epinephelus marginatus* juveniles fed different experimental diets in different temperatures (LT—lower temperature; HT—higher temperature).

Fatty Acid	Dietary FA Source/Temperature (Lower or Higher)								*p*-Values		
	D1-LT	D1-HT	D2-LT	D2-HT	D3-LT	D3-HT	D4-LT	D4-HT	T	D	D × T
12:0	0.45 ± 0.14	0.36 ± 0.07	0.44 ± 0.12	0.49 ± 0.07	1.86 ± 1.44	0.30 ± 0.08	0.23 ± 0.08	0.27 ± 0.08	0.264	0.330	0.270
14:0	0.55 ± 0.07 *	0.61 ± 0.05 **	0.67 ± 0.08 *	0.61 ± 0.05 **	1.12 ± 0.62 *	0.59 ± 0.04 **	0.53 ± 0.05 *	0.52 ± 0.05 **	<0.001	0.939	0.991
16:0	12.08 ± 0.60	12.53 ± 0.36	13.62 ± 0.97	12.06 ± 0.65	10.22 ± 1.27	13.57 ± 0.55	13.38 ± 1.71	12.66 ± 0.61	0.560	0.588	0.046
16:1	0.81 ± 0.06	0.93 ± 0.04	0.90 ± 0.08	0.80 ± 0.04	1.37 ± 0.40	0.84 ± 0.04	0.97 ± 0.09	0.82 ± 0.03	0.108	0.244	0.147
18:0	16.12 ± 0.88	16.56 ± 1.11	19.05 ± 1.38	17.95 ± 1.31	14.32 ± 2.01	15.66 ± 0.87	17.09 ± 2.26	16.02 ± 0.66	0.923	0.097	0.762
18:1n-9	20.46 ± 0.48	21.06 ± 0.80	23.99 ± 1.39	19.50 ± 0.33	17.83 ± 3.06	21.18 ± 0.54	22.78 ± 2.05	20.36 ± 0.68	0.458	0.358	0.037
18:1n-7	2.77 ± 0.16	2.41 ± 0.09	3.24 ± 0.18 *	2.34 ± 0.05 **	2.72 ± 0.12	2.38 ± 0.09	2.87 ± 0.23 *	2.25 ± 0.06 **	<0.001	0.268	0.142
18:2n-6	8.98 ± 0.23	8.39 ± 0.17	8.52 ± 0.14	8.06 ± 0.25	7.95 ± 0.86	8.02 ± 0.13	7.62 ± 0.65	8.02 ± 0.18	0.605	0.152	0.572
18:3n-3	0.47 ± 0.01	0.47 ± 0.01	0.41 ± 0.02	0.44 ± 0.02	0.78 ± 0.32	0.42 ± 0.01	0.38 ± 0.05	0.41 ± 0.01	0.333	0.262	0.207
18:4n-3	0.30 ± 0.03	0.31 ± 0.02	0.30 ± 0.01	0.58 ± 0.19	0.77 ± 0.51	0.50 ± 0.13	0.38 ± 0.09	0.33 ± 0.07	0.972	0.334	0.585
20:1n-9	1.13 ± 0.04	1.02 ± 0.06	1.32 ± 0.12	0.95 ± 0.04	1.93 ± 0.72	0.91 ± 0.04	1.34 ± 0.20	0.96 ± 0.03	0.155	0.719	0.308
20:2n-6	0.67 ± 0.03	0.60 ± 0.03	0.67 ± 0.03	0.79 ± 0.14	1.27 ± 0.62	0.59 ± 0.06	0.59 ± 0.07	0.59 ± 0.05	0.318	0.408	0.243
20:4n-6	5.90 ± 0.16 ^a^	6.03 ± 0.25	4.31 ± 0.48 ^b^*	5.69 ± 0.20 **	5.40 ± 0.18 ^ab^	5.49 ± 0.22	4.47 ± 0.73 ^a^*	5.57 ± 0.13 **	0.007	0.021	0.142
20:5n-3	4.74 ± 0.16 ^a^	4.38 ± 0.27 ^a^	3.13 ± 0.40 ^ab^	4.28 ± 0.78 ^a^	4.23 ± 0.67 ^ab^	3.21 ± 0.23 ^ab^	2.55 ± 0.59 ^b^	2.27 ± 0.10 ^b^	0.683	<0.001	0.115
22:5n-3	1.87 ± 0.11	1.74 ± 0.10	1.35 ± 0.18	1.64 ± 0.08	4.19 ± 2.38	1.56 ± 0.05	1.52 ± 0.27	1.51 ± 0.06	0.277	0.262	0.230
22:6n-3	22.69 ± 1.37	22.59 ± 1.63	18.08 ± 3.26	23.84 ± 1.17	24.06 ± 1.66	24.80 ± 1.21	23.29 ± 4.16	27.44 ± 1.55	0.093	0.210	0.493
∑SFA	29.20 ± 1.15	30.14 ± 1.39	33.78 ± 2.41	31.11 ± 1.86	27.51 ± 1.46	30.12 ± 1.15	31.24 ± 3.87	29.46 ± 1.06	0.987	0.294	0.563
∑MUFA	25.18 ± 0.62	25.02 ± 0.94	29.44 ± 1.71 *	23.58 ± 0.37 **	23.85 ± 1.95	25.31 ± 0.59	27.96 ± 2.53	24.39 ± 0.72	0.097	0.514	0.048
∑PUFA	45.62 ± 1.56	44.84 ± 2.06	36.78 ± 4.10	45.31 ± 1.80	48.63 ± 3.31	44.58 ± 1.62	40.81 ± 6.33	46.14 ± 1.75	0.461	0.344	0.219
∑LC-PUFA	35.88 ± 1.69	35.82 ± 2.15	27.54 ± 3.98	36.23 ± 1.67	39.14 ± 3.32	35.64 ± 1.60	32.42 ± 5.64	37.38 ± 1.78	0.379	0.324	0.215
∑n-3 PUFA	30.08 ± 1.61	29.88 ± 1.90	23.29 ± 3.57	30.78 ± 1.51	34.03 ± 3.44	30.48 ± 1.51	28.13 ± 4.94	31.96 ± 1.69	0.492	0.300	0.231
∑n-6 PUFA	15.55 ± 0.15 ^a^	14.96 ± 0.23	13.49 ± 0.59 ^ab^	14.53 ± 0.33	14.61 ± 0.37 ^ab^	14.09 ± 0.22	12.68 ± 1.42 ^b^	14.19 ± 0.15	0.430	0.026	0.234
DHA/EPA	4.76 ± 0.19 ^a^	5.28 ± 0.29 ^a^	6.04 ± 1.19 ^a^	6.22 ± 0.77 ^ab^	6.26 ± 0.73 ^a^	7.83 ± 0.23 ^b^	9.66 ± 1.04 ^b^	12.12 ± 0.63 ^c^	0.065	<0.001	0.640

Values represent means ± standard error of the mean (*n* = 3 animals per tank; *n* = 3 tanks by treatments). ^a,b,c^ Different letters indicate statistical differences between experimental diets by Tukey’s test (*p* < 0.05). *, ** Symbol indicates statistical differences within the same diet at different temperatures by Tukey’s test (*p* < 0.05). T represents *p*-value of temperature; D represents *p*-value of diet; and DxT represents *p*-value of diet versus temperature. ∑SFA, ∑MUFA, ∑PUFA, ∑LC-PUFA, ∑n-3 PUFA, and ∑n-6 PUFA are the sum of saturated (SFA), monosaturated (MUFA), polyunsaturated (PUFA), long-chain polyunsaturated fatty acids (LC-PUFA), polyunsaturated n3 (n-3 PUFA), and polyunsaturated n6 (n-6 PUFA), respectively. DHA/EPA is the ratio between the sum of total DHA (docosahexaenoic acid, 22:6n-3) and EPA (eicosapentaenoic acid, 20:5n-3).

**Table 7:** Fatty acid composition of triacylglycerol in the liver of *Epinephelus marginatus* juveniles fed different experimental diets in different temperatures (LT—lower temperature; HT—higher temperature).

Fatty Acid	Dietary FA Source/Temperature (Lower or Higher)								*p*-Values		
	D1-LT	D1-HT	D2-LT	D2-HT	D3-LT	D3-HT	D4-LT	D4-HT	T	D	D × T
12:0	1.62 ± 0.15	1.25 ± 0.16	1.73 ± 0.17	1.79 ± 0.41	2.42 ± 0.57	1.35 ± 0.06	1.71 ± 0.24 *	1.08 ± 0.06 **	0.017	0.267	0.289
14:0	3.98 ± 0.21 ^a^	3.61 ± 0.09	4.01 ± 0.14 ^b^*	4.52 ± 0.30 **	4.58 ± 0.29 ^a^	4.07 ± 0.07	4.40 ± 0.43 ^a^	4.00 ± 0.16	0.046	<0.001	<0.001
16:0	19.60 ± 0.35	19.59 ± 0.33 ^ab^	19.26 ± 0.24	19.22 ± 0.22 ^a^	18.87 ± 0.37	18.91 ± 0.26 ^a^	18.45 ± 0.25 *	20.24 ± 0.20 ^b^**	0.035	0.129	0.651
16:1	4.79 ± 0.13 *	4.21 ± 0.07 **	4.57 ± 0.09 *	4.28 ± 0.07 **	4.72 ± 0.17 *	4.23 ± 0.14 **	5.01 ± 0.12 *	4.36 ± 0.07 **	<0.001	0.116	0.438
18:0	4.37 ± 0.10 ^a^*	5.17 ± 0.20 **	4.17 ± 0.06 ^ab^*	4.77 ± 0.16 **	4.25 ± 0.23 ^ab^*	4.89 ± 0.22 **	3.70 ± 0.21 ^b^*	4.69 ± 0.14 **	<0.001	0.013	0.651
18:1n-9	36.73 ± 0.32	38.21 ± 0.42	35.75 ± 0.53	36.73 ± 0.45	35.86 ± 0.61	37.25 ± 0.42	36.32 ± 0.47	36.62 ± 0.34	0.139	0.002	0.571
18:1n-7	3.06 ± 0.04 *	2.88 ± 0.05 ^ab^**	2.97 ± 0.04	2.89 ± 0.03 ^b^	2.85 ± 0.07	2.80 ± 0.01 ^ab^	2.96 ± 0.09	2.75 ± 0.04 ^a^	0.001	0.029	0.355
18:2n-6	12.02 ± 0.37 *	10.89 ± 0.26 **	12.44 ± 0.39	12.26 ± 0.35	12.26 ± 0.29	12.04 ± 0.30	12.27 ± 0.51	11.86 ± 0.36	0.072	0.102	0.551
18:3n-3	1.17 ± 0.05 *	0.87 ± 0.02 ^a^**	1.28 ± 0.04 *	0.99 ± 0.03 ^b^ **	1.22 ± 0.04 *	0.97 ± 0.02 ^ab^**	0,95± 0.05 *	0.96 ± 0.03 ^ab^**	<0.001	0.033	0.493
18:4n-3	0.42 ± 0.03 ^a^*	0.31 ± 0.05 **	0.57 ± 0.03 ^b^*	0.30 ± 0.04 **	0.46 ± 0.04 ^ab^*	0.21 ± 0.01 **	0.35 ± 0.02 ^a^*	0.21 ± 0.03 **	<0.001	0.021	0.412
20:1n-9	2.52 ± 0.11	2.67 ± 0.04 ^a^	2.39 ± 0.14	2.31 ± 0.11 ^b^	2.15 ± 0.10	2.31 ± 0.08 ^b^	2.56 ± 0.19	2.38 ± 0.07 ^ab^	0.893	0.021	0.412
20:2n-6	0.82 ± 0.02 ^ab^	0.87 ± 0.02	0.85 ± 0.03 ^ab^	0.82 ± 0.03	0.78 ± 0.04 ^a^	0.81 ± 0.02	0.89 ± 0.04 ^b^	0.86 ± 0.01	0.750	0.025	0.258
20:4n-6	1.31 ± 0.03 *	1.57 ± 0.07 **	1.34 ± 0.05	1.45 ± 0.10	1.29 ± 0.04 *	1.61 ± 0.07 **	1.26 ± 0.04 *	1.48 ± 0.04 **	<0.001	0.493	0.321
20:5n-3	2.80 ± 0.09 ^a^	2.89 ± 0.10 ^a^	2.84 ± 0.20 ^a^	2.60 ± 0.09 ^a^	2.17 ± 0.12 ^b^*	2.48 ± 0.07 ^b^**	1.58 ± 0.10 ^c^	1.58 ± 0.04 ^c^	0.603	<0.001	0.097
22:5n-3	1.63 ± 0.06	1.67 ± 0.07 ^a^	1.72 ± 0.03 *	1.48 ± 0.06 ^ab^**	1.50 ± 0.09	1.47 ± 0.07 ^ab^	1.61 ± 0.06 *	1.38 ± 0.03 ^b^**	0.012	0.024	0.063
22:6n-3	3.15 ± 0.12 ^a^	3.35 ± 0.15 ^a^	4.10 ± 0.17 ^ab^	3.61 ± 0.17 ^ab^	4.64 ± 0.28 ^b^	4.60 ± 0.16 ^b^	5.70 ± 0.39 ^c^	5.54 ± 0.20 ^c^	0.455	<0.001	0.509
∑SFA	29.57 ± 0.33	29.62 ± 0.51	29.17 ± 0.25	30.30 ± 0.52	30.12 ± 0.80	29.22 ± 0.33	28.26 ± 0.53	30.01 ± 0.30	0.152	0.606	0.049
∑MUFA	47.10 ± 0.52	47.96 ± 0.46	45.68 ± 0.75	46.20 ± 0.58	45.57 ± 0.78	46.59 ± 0.55	46.84 ± 0.70	46.11 ± 0.36	0.339	0.051	0.463
∑PUFA	23.33 ± 0.61	22.42 ± 0.42 ^a^	25.15 ± 0.77 *	23.50 ± 0.31 ^ab^**	24.31 ± 0.58	24.19 ± 0.39 ^b^	24.90 ± 0.50	23.88 ± 0.38 ^b^	0.015	0.011	0.560
∑LC-PUFA	9.72 ± 0.27	10.35 ± 0.33	10.86 ± 0.36	9.96 ± 0.36	10.37 ± 0.42	10.98 ± 0.25	11.04 ± 0.54	10.85 ± 0.28	0.884	0.094	0.144
∑n-3 PUFA	9.18 ± 0.29 ^a^	9.09 ± 0.24 ^a^	10.51 ± 0.38 ^ab^	8.97 ± 0.22 ^b^	9.98 ± 0.38 ^ab^*	9.73 ± 0.22 ^ab^**	10.48 ± 0.48 ^b^	9.67 ± 0.24 ^b^	0.006	0.033	0.133
∑n-6 PUFA	14.15 ± 0.38	13.33 ± 0.28 ^a^	14.64 ± 0.41	14.53 ± 0.28 ^b^	14.33 ± 0.30	14.46 ± 0.32 ^ab^	14.42 ± 0.46 *	14.21 ± 0.35 ^c^**	0.001	<0.001	0.001
DHA/EPA	1.13 ± 0.04 ^a^	1.16 ± 0.02 ^a^	1.46 ± 0.04 ^a^	1.39 ± 0.04 ^b^	2.19 ± 0.21 ^b^	1.85 ± 0.06 ^c^	3.68 ± 0.27 ^c^	3.50 ± 0.09 ^d^	0.160	<0.001	0.582

Values represent means ± standard error of the mean (*n* = 3 animals per tank; *n* = 3 tanks by treatments). ^a,b,c,d^ Different letters indicate statistical differences between experimental diets by Tukey’s test (*p* < 0.05). *, ** Symbol indicates statistical differences within the same diet at different temperatures by Tukey’s test (*p* < 0.05). T represents *p*-value of temperature; D represents *p*-value of diet; and DxT represents *p*-value of diet versus temperature. ∑SFA, ∑MUFA, ∑PUFA, ∑LC-PUFA, ∑n-3 PUFA, and ∑n-6 PUFA are the sum of saturated (SFA), monosaturated (MUFA), polyunsaturated (PUFA), long-chain polyunsaturated fatty acids (LC-PUFA), polyunsaturated n3 (n-3 PUFA), and polyunsaturated n6 (n-6 PUFA), respectively. DHA/EPA is the ratio between the sum of total DHA (docosahexaenoic acid, 22:6n-3) and EPA (eicosapentaenoic acid, 20:5n-3).

**Table 8:** Fatty acid composition of phospholipids in the liver of *Epinephelus marginatus* juveniles fed different experimental diets in different temperatures (LT—lower temperature; HT—higher temperature).

Fatty Acid	Dietary FA Source/Temperature (Lower or Higher)								*p*-Values		
	D1-LT	D1-HT	D2-LT	D2-HT	D3-LT	D3-HT	D4-LT	D4-HT	T	D	D × T
12:0	0.72 ± 0.13	0.55 ± 0.09	0.70 ± 0.08	0.70 ± 0.13	0.81 ± 0.10	0.54 ± 0.07	0.61 ± 0.09	0.51 ± 0.08	0.053	0.569	0.690
14:0	1.57 ± 0.10	1.58 ± 0.10	2.06 ± 0.12	2.16 ± 0.48	1.79 ± 0.13	1.59 ± 0.14	1.83 ± 0.16	1.53 ± 0.12	0.400	0.116	0.855
16:0	14.85 ± 0.30	13.12 ± 0.40	16.11 ± 0.25	14.11 ± 0.80	15.49 ± 0.72 *	13.07 ± 0.74 **	15.93 ± 0.26 *	13.83 ± 0.59 **	<0.001	0.082	0.698
16:1	2.27 ± 0.10	2.40 ± 0.13	2.93 ± 0.35 *	2.23 ± 0.21 **	2.38 ± 0.25	2.13 ± 0.11	2.65 ± 0.19 *	2.01 ± 0.07 **	0.007	0.605	0.081
18:0	14.97 ± 0.64	16.15 ± 0.44	13.33 ± 0.47 *	16.17 ± 1.31 **	14.14 ± 1.16 *	16.91 ± 0.51 **	12.20 ± 0.57 *	16.62 ± 0.36 **	<0.001	0.314	0.206
18:1n-9	16.32 ± 0.59	19.34 ± 0.37	18.78 ± 0.84	17.22 ± 0.96	17.15 ± 1.85	16.97 ± 0.56	18.13 ± 0.89	15.95 ± 0.41	0.579	0.742	0.019
18:1n-7	2.18 ± 0.07	2.10 ± 0.02	2.23 ± 0.04 *	1.99 ± 0.08 **	2.15 ± 0.09 *	1.90 ± 0.03 **	2.29 ± 0.04 *	1.88 ± 0.03 **	<0.001	0.296	0.033
18:2n-6	11.26 ± 0.32	10.51 ± 0.21	11.76 ± 0.36	11.02 ± 0.51	11.91 ± 0.47 *	10.47 ± 0.28 **	12.56 ± 0.24 *	10.58 ± 0.26 **	<0.001	0.240	0.247
18:3n-3	0.97 ± 0.04 *	0.76 ± 0.04 **	1.04 ± 0.06 *	0.79 ± 0.07 **	1.00 ± 0.07 *	0.73 ± 0.03 **	1.01 ± 0.03 *	0.73 ± 0.04 **	<0.001	0.939	0.789
18:4n-3	2.83 ± 0.14	2.13 ± 0.73	2.14 ± 0.26	2.65 ± 0.42	2.86 ± 0.34	2.12 ± 0.56	1.86 ± 0.13	3.46 ± 0.70	0.429	0.977	0.029
20:1n-9	1.30 ± 0.10	1.42 ± 0.03	1.24 ± 0.06	1.14 ± 0.06	1.21 ± 0.07	1.24 ± 0.10	1.36 ± 0.10	1.12 ± 0.06	0.337	0.146	0.104
20:2n-6	0.90 ± 0.05	0.87 ± 0.02	1.02 ± 0.13	0.78 ± 0.03	0.85 ± 0.04	0.80 ± 0.03	0.97 ± 0.05	1.11 ± 0.21	0.543	0.141	0.298
20:4n-6	6.80 ± 0.13	7.07 ± 0.19	5.77 ± 0.32 *	7.32 ± 0.39 **	6.17 ± 0.47 *	7.79 ± 0.26 **	6.03 ± 0.17 *	7.47 ± 0.40 **	<0.001	0.344	0.154
20:5n-3	6.66 ± 0.09 ^a^	6.21 ± 0.38 ^a^	5.73 ± 0.39 ^b^	5.34 ± 0.30 ^ab^	4.80 ± 0.24 ^c^	4.99 ± 0.22 ^b^	3.69 ± 0.27 ^d^	3.84 ± 0.20 ^c^	0.590	<0.001	0.563
22:5n-3	1.58 ± 0.06 ^a^	1.61 ± 0.06 ^a^	1.43 ± 0.03 ^ab^	1.31 ± 0.05 ^b^	1.36 ± 0.06 ^b^	1.31 ± 0.05 ^bc^	1.40 ± 0.04 ^ab^	1.09 ± 0.07 ^c^	0.030	<0.001	0.029
22:6n-3	14.83 ± 0.40 ^ab^	14.17 ± 0.43 ^a^	13.72 ± 0.87 ^a^	15.08 ± 0.64 ^ab^	15.94 ± 1.18 ^ab^	17.44 ± 0.59 ^bc^	17.49 ± 0.82 ^b^	18.29 ± 0.98 ^c^	0.214	<0.001	0.542
∑SFA	32.11 ± 0.84	31.40 ± 0.87	32.21 ± 0.56	33.14 ± 1.37	32.23 ± 1.33	32.11 ± 0.97	30.57 ± 0.28	32.49 ± 0.65	0.437	0.605	0.481
∑MUFA	22.07 ± 0.80	25.26 ± 0.51	25.18 ± 1.14	22.58 ± 1.26	22.89 ± 2.18	22.25 ± 0.70	24.43 ± 1.13	20.95 ± 0.50	0.198	0.709	0.016
∑PUFA	45.82 ± 0.59	43.34 ± 1.07	42.61 ± 1.28	44.28 ± 1.54	44.88 ± 1.53	45.64 ± 1.23	45.00 ± 1.08	46.57 ± 0.54	0.669	0.197	0.239
∑LC-PUFA	30.77 ± 0.55	29.94 ± 0.81	27.66 ± 1.26	29.82 ± 1.23	29.12 ± 1.82	32.32 ± 1.03	29.57 ± 1.12	31.79 ± 1.11	0.058	0.235	0.365
∑n-3 PUFA	26.87 ± 0.52	24.88 ± 0.89	24.07 ± 1.03	25.16 ± 1.15	25.96 ± 1.16	26.58 ± 1.01	25.45 ± 1.07	27.41 ± 0.34	0.480	0.256	0.161
∑n-6 PUFA	18.95 ± 0.32	18.45 ± 0.22	18.54 ± 0.40	19.12 ± 0.57	18.93 ± 0.57	19.06 ± 0.34	19.56 ± 0.14	19.15 ± 0.36	0.864	0.358	0.473
DHA/EPA	2.23 ± 0.07 ^a^	2.36 ± 0.20 ^a^	2.48 ± 0.25 ^ab^	2.88 ± 0.18 ^ab^	4.85 ± 0.22 ^b^	3.51 ± 0.09 ^b^	4.85 ± 0.30 ^c^	4.90 ± 0.41 ^c^	0.275	<0.001	0897

Values represent means ± standard error of the mean (*n* = 3 animals per tank; *n* = 3 tanks by treatments). ^a,b,c,d^ Different letters indicate statistical differences between experimental diets by Tukey’s test (*p* < 0.05). *, ** Symbol indicates statistical differences within the same diet at different temperatures by Tukey’s test (*p* < 0.05). T represents *p*-value of temperature; D represents *p*-value of diet; and DxT represents *p*-value of diet versus temperature. ∑SFA, ∑MUFA, ∑PUFA, ∑LC-PUFA, ∑n-3 PUFA, and ∑n-6 PUFA are the sum of saturated (SFA), monosaturated (MUFA), polyunsaturated (PUFA), long-chain polyunsaturated fatty acids (LC-PUFA), polyunsaturated n3 (n-3 PUFA), and polyunsaturated n6 (n-6 PUFA), respectively. DHA/EPA is the ratio between the sum of total DHA (docosahexaenoic acid, 22:6n-3) and EPA (eicosapentaenoic acid, 20:5n-3).

**Table 9:** Fatty acid composition of triacylglycerol in the eyes of *Epinephelus marginatus* juveniles fed different experimental diets in different temperatures (LT—lower temperature; HT—higher temperature).

Fatty Acid	Dietary FA Source/Temperature (Lower or Higher)								*p*-Values		
	D1-LT	D1-HT	D2-LT	D2-HT	D3-LT	D3-HT	D4-LT	D4-HT	T	D	D × T
12:0	7.22 ± 0.17	6.61 ± 0.22	7.07 ± 0.24	7.22 ± 0.31	6.73 ± 0.21	6.98 ± 0.30	7.61 ± 0.21 *	6.01 ± 0.19 **	0.009	0.517	<0.001
14:0	5.66 ± 0.12	5.42 ± 0.13	5.55 ± 0.13	5.79 ± 0.13	5.51 ± 0.11	5.80 ± 0.19	6.30 ± 0.10	5.43 ± 0.10	0.115	0.091	<0.001
16:0	16.54 ± 0.12	16.98 ± 0.10	16.71 ± 0.12	16.77 ± 0.15	16.56 ± 0.13	17.04 ± 0.11	17.35 ± 0.73	17.07 ± 0.15	0.420	0.349	0.557
16:1	3.61 ± 0.06 ^a^	3.61 ± 0.03	3.64 ± 0.03 ^a^	3.55 ± 0.04	3.67 ± 0.02 ^a^	3.64 ± 0.03	4.02 ± 0.14 ^b^*	3.67 ± 0.06 **	0.013	<0.001	0.033
18:0	11.76 ± 1.49 *	7.90 ± 0.91 **	13.49 ± 1.80 *	7.41 ± 0.73 **	9.38 ± 1.31	6.97 ± 0.84	14.15 ± 1.56 *	8.09 ± 1.24 **	<0.001	0.120	0.395
18:1n-9	29.47 ± 1.39 *	33.89 ± 0.85 **	27.85 ± 1.68 *	34.03 ± 0.75 **	31.60 ± 1.31	34.11 ± 0.78	29.18 ± 2.66	33.03 ± 1.11	<0.001	0.553	0.667
18:1n-7	1.48 ± 0.12	1.77 ± 0.12	1.27 ± 0.13 *	1.78 ± 0.12 **	1.69 ± 0.18	1.86 ± 0.13	1.32 ± 0.16 *	1.76 ± 0.18 **	0.001	0.312	0.676
18:2n-6	10.74 ± 0.11	10.93 ± 0.14	10.80 ± 0.13	10.54 ± 0.15	10.86 ± 0.13	10.66 ± 0.17	9.17 ± 0.71 *	11.16 ± 0.15 **	0.049	0.117	<0.001
18:3n-3	1.21 ± 0.03 ^a^	1.23 ± 0.02	1.24 ± 0.02 ^a^	1.16 ± 0.02	1.18 ± 0.03 ^a^	1.16 ± 0.03	0.90 ± 0.13 ^b^	1.24 ± 0.03	0.128	0.039	0.001
18:4n-3	0.25 ± 0.02 ^a^	0.26 ± 0.02	0.24 ± 0.02 ^a^	0.27 ± 0.01	0.22 ± 0.01 ^ab^	0.24 ± 0.01	0.16 ± 0.02 ^b^*	0.22 ± 0.01 **	0.008	<0.001	0.463
20:1n-9	1.49 ± 0.02	1.51 ± 0.02	1.48 ± 0.02	1.44 ± 0.02	1.47 ± 0.02	1.44 ± 0.03	1.54 ± 0.10	1.43 ± 0.01	0.189	0.745	0.500
20:2n-6	0.50 ± 0.02	0.50 ± 0.01	0.51 ± 0.04	0.46 ± 0.01	0.50 ± 0.01	0.46 ± 0.02	0.44 ± 0.04	0.50 ± 0.01	0.531	0.619	0.066
20:4n-6	1.15 ± 0.02 ^a^	1.15 ± 0.03	1.19 ± 0.05 ^a^	1.10 ± 0.02	1.19 ± 0.03 ^a^	1.11 ± 0.04	0.81 ± 0.15 ^b^	1.16 ± 0.02	0.292	0.024	0.001
20:5n-3	3.06 ± 0.04	3.11 ± 0.03	2.80 ± 0.04	2.79 ± 0.04	2.47 ± 0.04	2.47 ± 0.03	1.39 ± 0.29	1.78 ± 0.04	0.186	<0.001	0.241
22:5n-3	1.02 ± 0.02	1.05 ± 0.01	1.01 ± 0.02	1.06 ± 0.02	1.03 ± 0.02	1.05 ± 0.02	0.83 ± 0.16	1.10 ± 0.03	0.052	0.594	0.168
22:6n-3	4.84 ± 0.27	4.10 ± 0.11 ^a^	5.15 ± 0.22	4.63 ± 0.13 ^a^	5.94 ± 0.24	5.01 ± 0.12 ^ab^	4.83 ± 1.01	6.36 ± 0.24 ^b^	0.590	0.038	0.016
∑SFA	41.18 ± 1.57 ^a^*	36.91 ± 1.06 **	42.82 ± 2.07 ^ab^*	37.20 ± 0.83 **	38.19 ± 1.49 ^a^	36.80 ± 1.03	45.42 ± 1.35 ^b^*	36.59 ± 1.23 **	<0.001	0.046	0.050
∑MUFA	36.05 ± 1.49	40.77 ± 0.97	34.25 ± 1.83 *	40.80 ± 0.83 **	38.43 ± 1.48	41.05 ± 0.89	36.06 ± 3.03	39.88 ± 1.22	<0.001	0.570	0.689
∑PUFA	22.77 ± 0.22	22.32 ± 0.20	22.93 ± 0.33	22.00 ± 0.19	23.38 ± 0.24	22.14 ± 0.28	18.53 ± 2.48	23.53 ± 0.38	0.391	0.260	0.005
∑LC-PUFA	10.57 ± 0.26	9.91 ± 0.13	10.66 ± 0.29	10.03 ± 0.14	11.12 ± 0.27	10.09 ± 0.16	8.29 ± 1.63	10.91 ± 0.30	0.872	0.456	0.020
∑n-3 PUFA	10.38 ± 0.22	9.75 ± 0.12	10.43 ± 0.24	9.91 ± 0.14	10.84 ± 0.24	9.92 ± 0.14	8.11 ± 1.61	10.70 ± 0.28	0.755	0.449	0.022
∑n-6 PUFA	12.39 ± 0.13	12.58 ± 0.18	12.50 ± 0.19	12.10 ± 0.18	12.54 ± 0.15	12.22 ± 0.21	10.42 ± 0.88	12.83 ± 0.17	0.083	0.092	<0.001
DHA/EPA	1.59 ± 0.11 ^a^	1.32 ± 0.04 ^a^	1.84 ± 0.08 ^a^*	1.66 ± 0.05 ^a^**	2.41 ± 0.10 ^b^	2.03 ± 0.06 ^b^	3.52 ± 0.16 ^c^	3.57 ± 0.14 ^c^	0.009	<0.001	0.186

Values represent means ± standard error of the mean (*n* = 3 animals per tank; *n* = 3 tanks by treatments). ^a,b,c^ Different letters indicate statistical differences between experimental diets by Tukey’s test (*p* < 0.05). *, ** Symbol indicates statistical differences within the same diet at different temperatures by Tukey’s test (*p* < 0.05). T represents *p*-value of temperature; D represents *p*-value of diet; and DxT represents *p*-value of diet versus temperature. ∑SFA, ∑MUFA, ∑PUFA, ∑LC-PUFA, ∑n-3 PUFA, and ∑n-6 PUFA are the sum of saturated (SFA), monosaturated (MUFA), polyunsaturated (PUFA), long-chain polyunsaturated fatty acids (LC-PUFA), polyunsaturated n3 (n-3 PUFA), and polyunsaturated n6 (n-6 PUFA), respectively. DHA/EPA is the ratio between the sum of total DHA (docosahexaenoic acid, 22:6n-3) and EPA (eicosapentaenoic acid, 20:5n-3).

**Table 10:** Fatty acid composition of phospholipids in the eyes of *Epinephelus marginatus* juveniles fed different experimental diets in different temperatures (LT—lower temperature; HT—higher temperature).

Fatty Acid	Dietary FA Source/Temperature (Lower or Higher)								*p*-Values		
	D1-LT	D1-HT	D2-LT	D2-HT	D3-LT	D3-HT	D4-LT	D4-HT	T	D	D × T
12:0	0.68 ± 0.14	1.23 ± 0.47	0.90 ± 0.31	0.93 ± 0.19	0.61 ± 0.09	0.51 ± 0.08	0.52 ± 0.11	0.74 ± 0.19	0.316	0.253	0.549
14:0	1.88 ± 0.08	2.03 ± 0.33	1.96 ± 0.26	1.83 ± 0.18	1.64 ± 0.11	1.55 ± 0.11	1.85 ± 0.13	1.75 ± 0.15	0.753	0.255	0.869
16:0	22.63 ± 1.14	19.89 ± 1.28	21.71 ± 1.54	19.95 ± 1.13	21.26 ± 1.03	19.27 ± 1.06	26.67 ± 1.16 *	19.34 ± 0.85 **	<0.001	0.171	0.110
16:1	2.71 ± 0.10	2.56 ± 0.16	2.76 ± 0.09	2.65 ± 0.13	2.97 ± 0.13 *	2.46 ± 0.04 **	2.61 ± 0.09	2.56 ± 0.04	0.013	0.626	0.169
18:0	26.88 ± 1.09	24.28 ± 1.48	26.52 ± 1.60	24.38 ± 1.42	28.36 ± 1.53 *	22.73 ± 0.58 **	26.89 ± 1.61	23.57 ± 0.85	0.001	0.995	0.592
18:1n-9	27.05 ± 1.23 *	30.97 ± 1.05 **	27.30 ± 1.33 *	31.02 ± 0.54 **	26.39 ± 1.06 *	30.03 ± 0.88 **	24.92 ± 1.33 *	31.68 ± 0.68 **	<0.001	0.771	0.485
18:1n-7	2.88 ± 0.10	2.95 ± 0.07	2.84 ± 0.11	3.06 ± 0.13	2.85 ± 0.18	3.14 ± 0.10	2.73 ± 0.17	2.93 ± 0.08	0.360	0.788	0.670
18:2n-6	1.79 ± 0.51	3.07 ± 0.60	1.64 ± 0.40	2.85 ± 0.37	1.81 ± 0.37 *	4.10 ± 0.53 **	1.43 ± 0.37	2.57 ± 0.50	<0.001	0.233	0.567
18:3n-3	0.21 ± 0.04	0.19 ± 0.03	0.22 ± 0.05	0.33 ± 0.08	0.32 ± 0.05	0.23 ± 0.05	0.17 ± 0.04	0.21 ± 0.02	0.751	0.163	0.191
18:4n-3	0.67 ± 0.17	0.37 ± 0.10	0.87 ± 0.16	0.62 ± 0.20	1.15 ± 0.27 *	0.24 ± 0.06 **	0.79 ± 0.20	0.62 ± 0.18	0.004	0.620	0.210
20:1n-9	2.71 ± 0.16 *	2.33 ± 0.13 **	2.76 ± 0.11 *	2.12 ± 0.07 **	2.59 ± 0.13 *	2.03 ± 0.11 **	2.71 ± 0.11	2.41 ± 0.20	<0.001	0.296	0.605
20:2n-6	0.43 ± 0.05	0.50 ± 0.06	0.45 ± 0.05	0.55 ± 0.05	0.59 ± 0.05	0.65 ± 0.06	0.44 ± 0.05	0.50 ± 0.05	0.121	0.389	0.090
20:4n-6	0.51 ± 0.20	1.95 ± 0.79 ^a^	0.95 ± 0.59	0.98 ± 0.47 ^a^	0.81 ± 0.32 *	4.74 ± 1.51 ^b^**	0.34 ± 0.05	1.27 ± 0.52 ^a^	0.001	0.014	0.026
20:5n-3	0.72 ± 0.09	0.64 ± 0.07 ^a^	0.79 ± 0.08	0.62 ± 0.04 ^a^	0.83 ± 0.23	1.50 ± 0.48 ^b^	0.44 ± 0.05	0.79 ± 0.09 ^ab^	0.164	0.028	0.108
22:5n-3	1.87 ± 0.25	1.43 ± 0.27	1.93 ± 0.19	1.80 ± 0.24	1.92 ± 0.21	1.31 ± 0.41	2.28 ± 0.29	2.15 ± 0.10	0.081	0.135	0.761
22:6n-3	6.38 ± 0.44	5.63 ± 0.55	6.41 ± 0.43	6.30 ± 0.35	5.89 ± 0.53	5.48 ± 1.01	5.19 ± 0.35	6.91 ± 0.38	0.732	0.662	0.138
∑SFA	52.07 ± 1.89	47.43 ± 1.53	51.09 ± 1.96	47.10 ± 0.89	51.87 ± 2.02 *	44.09 ± 1.58 **	55.94 ± 1.93 *	45.40 ± 1.15 **	<0.001	0.514	0.269
∑MUFA	35.35 ± 1.27 *	38.80 ± 1.20 **	35.64 ± 1.41	38.85 ± 0.55	34.81 ± 1.18	37.66 ± 0.96	32.97 ± 1.55 *	39.58 ± 0.62 **	<0.001	0.767	0.430
∑PUFA	12.58 ± 0.85	13.77 ± 1.15	13.27 ± 0.99	14.04 ± 0.49	13.32 ± 1.33 *	18.24 ± 2.32 **	11.08 ± 0.62 *	15.02 ± 0.94 **	0.003	0.095	0.245
∑LC-PUFA	9.91 ± 0.62	10.15 ± 1.05	10.53 ± 0.77	10.25 ± 0.33	10.04 ± 1.00 *	13.67 ± 2.08 **	8.69 ± 0.49	11.62 ± 0.64	0.025	0.226	0.137
∑n-3 PUFA	9.85 ± 0.60	8.26 ± 0.91	10.23 ± 0.73	9.67 ± 0.63	10.10 ± 1.06	8.76 ± 1.25	8.87 ± 0.60	10.68 ± 0.60	0.495	0.732	0.231
∑n-6 PUFA	2.73 ± 0.73	5.52 ± 1.26 ^ab^	3.04 ± 0.97	4.37 ± 0.87 ^a^	3.22 ± 0.63 *	9.48 ± 2.01 ^b^**	2.21 ± 0.41	4.34 ± 1.01 ^a^	<0.001	0.023	0.098
DHA/EPA	9.73 ± 1.34	8.96 ± 0.26	8.41 ± 0.72	10.20 ± 0.58	10.07 ± 1.81	7.11 ± 3.22	12.59 ± 1.79	9.01 ± 0.64	0.216	0.580	0.323

Values represent means ± standard error of the mean (*n* = 3 animals per tank; *n* = 3 tanks by treatments). ^a,b^ Different letters indicate statistical differences between experimental diets by Tukey’s test (*p* < 0.05). *, ** Symbol indicates statistical differences within the same diet at different temperatures by Tukey’s test (*p* < 0.05). T represents *p*-value of temperature; D represents *p*-value of diet; and DxT represents *p*-value of diet versus temperature. ∑SFA, ∑MUFA, ∑PUFA, ∑LC-PUFA, ∑n-3 PUFA, and ∑n-6 PUFA are the sum of saturated (SFA), monosaturated (MUFA), polyunsaturated (PUFA), long-chain polyunsaturated fatty acids (LC-PUFA), polyunsaturated n3 (n-3 PUFA), and polyunsaturated n6 (n-6 PUFA), respectively. DHA/EPA is the ratio between the sum of total DHA (docosahexaenoic acid, 22:6n-3) and EPA (eicosapentaenoic acid, 20:5n-3).

**Table 11:** Fatty acid composition of triacylglycerol in the brain of *Epinephelus marginatus* juveniles fed different experimental diets in different temperatures (LT—lower temperature; HT—higher temperature).

Fatty Acid	Dietary FA Source/Temperature (Lower or Higher)								*p*-Values		
	D1-LT	D1-HT	D2-LT	D2-HT	D3-LT	D3-HT	D4-LT	D4-HT	T	D	D × T
12:0	5.25 ± 0.41	4.86 ± 0.81	5.17 ± 0.45	5.40 ± 0.44	5.25 ± 0.45	3.68 ± 0.60	6.07 ± 0.48 *	4.10 ± 0.81 **	0.022	0.504	0.159
14:0	4.41 ± 0.33	4.67 ± 0.50	4.81 ± 0.23	4.70 ± 0.23	4.89 ± 0.24	4.92 ± 0.15	5.35 ± 0.17	4.63 ± 0.41	0.521	0.464	0.409
16:0	15.17 ± 1.05 *	18.97 ± 0.49 ^a^**	15.65 ± 0.29	15.62 ± 0.25 ^b^	15.18 ± 0.41	16.54 ± 0.44 ^ab^	15.12 ± 0.32	15.83 ± 0.76 ^b^	<0.001	0.033	0.010
16:1	3.15 ± 0.17	3.28 ± 0.18	3.36 ± 0.06	3.13 ± 0.08	3.34 ± 0.10	3.34 ± 0.09	3.54 ± 0.06	3.32 ± 0.16	0.324	0.255	0.343
18:0	11.23 ± 1.58 *	17.15 ± 3.20 ^a^**	8.27 ± 0.64	11.41 ± 1.90 ^b^	8.33 ± 0.58	9.69 ± 0.89 ^b^	7.06 ± 0.41	8.84 ± 1.28 ^b^	0.006	<0.001	0.444
18:1n-9	31.20 ± 1.41 *	35.57 ± 1.04 **	33.10 ± 0.58	33.07 ± 0.32	32.67 ± 0.65	34.33 ± 0.71	33.37 ± 0.40	32.75 ± 1.14	0.032	0.945	0.025
18:1n-7	2.44 ± 0.09	2.56 ± 0.12	2.57 ± 0.04	2.36 ± 0.05	2.46 ± 0.04	2.46 ± 0.07	2.40 ± 0.05	2.32 ± 0.08	0.394	0.241	0.117
18:2n-6	7.85 ± 0.90 *	4.89 ± 1.36 ^a^**	9.49 ± 0.27	8.62 ± 0.71 ^b^	9.30 ± 0.26	9.29 ± 0.48 ^b^	9.50 ± 0.20	9.84 ± 0.47 ^b^	0.070	<0.001	0.078
18:3n-3	0.80 ± 0.09	0.48 ± 0.11 ^a^	0.86 ± 0.06	0.86 ± 0.09 ^b^	0.87 ± 0.04	0.89 ± 0.07 ^b^	0.93 ± 0.04	1.02 ± 0.06 ^b^	0.331	<0.001	0.039
18:4n-3	0.43 ± 0.05	0.47 ± 0.08	0.53 ± 0.13	0.34 ± 0.04	0.49 ± 0.08	0.27 ± 0.05	0.31 ± 0.04	0.31 ± 0.04	0.091	0.243	0.215
20:1n-9	1.47 ± 0.07	1.71 ± 0.08 ^a^	1.46 ± 0.03	1.36 ± 0.04 ^b^	1.38 ± 0.03	1.35 ± 0.04 ^b^	1.34 ± 0.03	1.32 ± 0.04 ^b^	0.314	<0.001	<0.001
20:2n-6	0.43 ± 0.01	0.39 ± 0.04	0.60 ± 0.13	0.46 ± 0.03	0.55 ± 0.06	0.45 ± 0.04	0.50 ± 0.08	0.44 ± 0.02	0.088	0.356	0.875
20:4n-6	2.20 ± 0.42 *	0.56 ± 0.23 ^a^**	1.93 ± 0.17	1.77 ± 0.15 ^b^	2.03 ± 0.14	1.82 ± 0.27 ^b^	1.84 ± 0.16	1.99 ± 0.28 ^b^	0.011	0.099	0.003
20:5n-3	2.30 ± 0.30 *	0.81 ± 0.45 ^a^**	2.60 ± 0.21	2.09 ± 0.29 ^b^	2.26 ± 0.11	2.04 ± 0.27 ^b^	1.94 ± 0.13	1.57 ± 0.07 ^ab^	<0.001	0.010	0.068
22:5n-3	1.56 ± 0.25 *	0.95 ± 0.27 **	1.16 ± 0.08	0.99 ± 0.11	1.18 ± 0.05	0.90 ± 0.10	1.45 ± 0.06	1.32 ± 0.08	0.007	0.087	0.378
22:6n-3	10.11 ± 2.21 *	2.65 ± 0.63 ^a^**	8.44 ± 0.94	7.83 ± 0.62 ^b^	9.82 ± 1.04	8.03 ± 1.30 ^b^	9.28 ± 0.63	10.38 ± 1.77 ^b^	0.020	0.068	0.011
∑SFA	36.06 ± 1.94 *	45.65 ± 3.02 ^a^**	33.90 ± 0.33	37.13 ± 1.56 ^b^	33.65 ± 0.50	34.82 ± 1.65 ^b^	33.60 ± 0.49	33.41 ± 0.80 ^b^	0.002	<0.001	0.009
∑MUFA	38.26 ± 1.70	43.16 ± 1.25	40.50 ± 0.64	39.92 ± 0.34	39.85 ± 0.77	41.48 ± 0.87	40.65 ± 0.47	39.71 ± 1.39	0.091	0.926	0.023
∑PUFA	25.68 ± 3.06 *	11.20 ± 2.71 ^a^**	25.60 ± 0.91	22.96 ± 1.66 ^b^	26.51 ± 1.02	23.70 ± 2.22 ^b^	25.75 ± 0.68	26.88 ± 1.60^b^	<0.001	<0.001	<0.001
∑LC-PUFA	16.60 ± 2.92 *	5.36 ± 1.50 **	14.72 ± 1.11	13.13 ± 1.02	15.84 ± 1.28	13.25 ± 1.86	15.01 ± 0.75	15.70 ± 2.01	0.004	0.073	0.006
∑n-3 PUFA	15.20 ± 2.50 *	5.36 ± 1.41 **	13.58 ± 0.95	12.12 ± 0.94	14.63 ± 1.14	12.13 ± 1.72	13.92 ± 0.62	14.61 ± 1.71	0.003	0.062	0.006
∑n-6 PUFA	10.48 ± 1.03 *	5.84 ± 1.59 ^a^**	12.02 ± 0.21	10.84 ± 0.80 ^b^	11.88 ± 0.17	11.56 ± 0.65 ^b^	11.83 ± 0.23	12.28 ± 0.34 ^b^	0.010	<0.001	0.008
DHA/EPA	4.87 ± 0.95	6.14 ± 2.12	3.41 ± 0.46	4.82 ± 1.26	4.32 ± 0.37	3.85 ± 0.34	4.95 ± 0.46	6.92 ± 1.53	0.168	0.195	0.710

Values represent means ± standard error of the mean (*n* = 3 animals per tank; *n* = 3 tanks by treatments). ^a,b^ Different letters indicate statistical differences between experimental diets by Tukey’s test (*p* < 0.05). *, ** Symbol indicates statistical differences within the same diet at different temperatures by Tukey’s test (*p* < 0.05). T represents *p*-value of temperature; D represents *p*-value of diet; and DxT represents *p*-value of diet versus temperature. ∑SFA, ∑MUFA, ∑PUFA, ∑LC-PUFA, ∑n-3 PUFA, and ∑n-6 PUFA are the sum of saturated (SFA), monosaturated (MUFA), polyunsaturated (PUFA), long-chain polyunsaturated fatty acids (LC-PUFA), polyunsaturated n3 (n-3 PUFA), and polyunsaturated n6 (n-6 PUFA), respectively. DHA/EPA is the ratio between the sum of total DHA (docosahexaenoic acid, 22:6n-3) and EPA (eicosapentaenoic acid, 20:5n-3).

**Table 12:** Fatty acid composition of phospholipids in the brain of *Epinephelus marginatus* juveniles fed different experimental diets in different temperatures (LT—lower temperature; HT—higher temperature).

Fatty Acid	Dietary FA Source/Temperature (Lower or Higher)								*p*-Values		
	D1-LT	D1-HT	D2-LT	D2-HT	D3-LT	D3-HT	D4-LT	D4-HT	T	D	D × T
12:0	0.15 ± 0.11	0.59 ± 0.24	1.15 ± 0.94	0.15 ± 0.03	0.14 ± 0.02	0.37 ± 0.10	0.23 ± 0.09	0.19 ± 0.04	0.586	0.279	0.027
14:0	0.59 ± 0.09	0.90 ± 0.21 ^a^	0.44 ± 0.03	0.43 ± 0.05 ^b^	0.45 ± 0.02	0.66 ± 0.11 ^ab^	0.53 ± 0.11	0.49 ± 0.05 ^b^	0.103	0.029	0.244
16:0	17.15 ± 0.88	18.63 ± 0.80	15.37 ± 0.77	16.62 ± 1.08	15.13 ± 0.35	16.15 ± 1.62	15.31 ± 1.28	14.61 ± 0.92	0.319	0.050	0.731
16:1	1.87 ± 0.08	2.06 ± 0.10	1.74 ± 0.08	1.77 ± 0.04	1.86 ± 0.05	1.87 ± 0.16	1.86 ± 0.15	1.69 ± 0.08	0.853	0.171	0.378
18:0	23.55 ± 1.12	20.46 ± 1.28	25.31 ± 0.48	23.80 ± 1.93	27.19 ± 0.87	22.93 ± 2.43	23.52 ± 1.72	27.09 ± 2.12	0.282	0.202	0.110
18:1n-9	30.06 ± 0.67 *	33.20 ± 0.62 **	29.22 ± 0.25	31.51 ± 0.56	28.47 ± 0.64	30.83 ± 1.54	28.12 ± 1.37	29.55 ± 1.11	0.033	0.108	0.316
18:1n-7	1.61 ± 0.03	1.64 ± 0.05	1.59 ± 0.06	1.49 ± 0.03	1.59 ± 0.03	1.51 ± 0.07	1.61 ± 0.06 *	1.39 ± 0.06 **	0.014	0.115	0.152
18:2n-6	0.94 ± 0.06	0.92 ± 0.12	1.19 ± 0.14	1.17 ± 0.21	0.79 ± 0.09	0.93 ± 0.10	1.08 ± 0.15	0.79 ± 0.16	0.624	0.138	0.440
18:3n-3	0.51 ± 0.07	0.59 ± 0.09	0.58 ± 0.10	0.35 ± 0.07	0.80 ± 0.10 *	0.46 ± 0.09 **	0.49 ± 0.08	0.56 ± 0.09	0.021	0.321	0.039
18:4n-3	0.61 ± 0.10	0.70 ± 0.23	0.70 ± 0.09	0.41 ± 0.09	0.78 ± 0.09	0.66 ± 0.17	0.62 ± 0.12	0.71 ± 0.14	0.544	0.677	0.462
20:1n-9	1.66 ± 0.10	1.74 ± 0.08	1.52 ± 0.09	1.38 ± 0.09	1.66 ± 0.08	1.50 ± 0.14	1.50 ± 0.14	1.46 ± 0.10	0.397	0.114	0.658
20:2n-6	0.25 ± 0.04 ^a^	0.23 ± 0.05	0.24 ± 0.02 ^a^	0.32 ± 0.03	0.43 ± 0.03 ^b^	0.29 ± 0.04	0.39 ± 0.03 ^b^	0.28 ± 0.05	0.087	0.011	0.021
20:4n-6	0.96 ± 0.23	0.21 ± 0.04	1.55 ± 0.34	2.55 ± 0.94	0.98 ± 0.18	0.99 ± 0.47	2.04 ± 0.76	1.07 ± 0.36	0.652	0.067	0.326
20:5n-3	1.10 ± 0.06	1.10 ± 0.06	0.97 ± 0.04	0.90 ± 0.05	1.06 ± 0.4	0.97 ± 0.09	1.18 ± 0.10 *	0.93 ± 0.08 **	0.042	0.150	0.358
22:5n-3	7.81 ± 0.94	4.57 ± 0.16	8.40 ± 0.76	5.67 ± 1.35	7.72 ± 0.91	8.92 ± 3.26	10.65 ± 3.62	8.92 ± 1.82	0.278	0.349	0.694
22:6n-3	11.17 ± 0.32	12.47 ± 0.40	10.03 ± 0.66	11.47 ± 0.29	10.96 ± 0.25	10.96 ± 0.67	10.87 ± 0.50	10.28 ± 0.97	0.155	0.104	0.174
∑SFA	41.45 ± 0.47	40.58 ± 0.54	42.28 ± 0.64	41.01 ± 1.10	42.90 ± 1.11	40.12 ± 2.03	41.42 ± 0.59	42.38 ± 1.60	0.249	0.902	0.459
∑MUFA	35.21 ± 0.83 *	38.64 ± 0.80 **	34.07 ± 0.34	36.16 ± 0.53	33.58 ± 0.65	35.70 ± 1.80	34.33 ± 0.92	34.09 ± 1.27	0.014	0.052	0.352
∑PUFA	23.34 ± 0.86	20.78 ± 0.53	23.65 ± 0.82	22.83 ± 0.93	23.51 ± 1.02	24.18 ± 3.02	24.26 ± 0.73	23.54 ± 1.82	0.421	0.576	0.742
∑LC-PUFA	21.29 ± 0.84	18.58 ± 0.53	21.18 ± 0.63	20.91 ± 0.90	21.14 ± 1.02	22.13 ± 3.07	25.14 ± 3.62	21.48 ± 1.84	0.326	0.409	0.623
∑n-3 PUFA	21.20 ± 0.66	19.42 ± 0.58	20.67 ± 0.52	18.80 ± 1.45	21.32 ± 0.84	21.97 ± 2.51	21.13 ± 0.96	21.40 ± 1.41	0.484	0.496	0.698
∑n-6 PUFA	2.14 ± 0.24	1.36 ± 0.12	2.98 ± 0.43	4.04 ± 1.11	2.20 ± 0.22	2.21 ± 0.55	3.13 ± 0.75	2.13 ± 0.48	0.693	0.062	0.403
DHA/EPA	10.25 ± 0.40	11.53 ± 0.63	10.37 ± 0.71 *	12.88 ± 0.50 **	10.40 ± 0.28	11.71 ± 0.68	10.18 ± 0.51	11.32 ± 1.20	0.001	0.577	0.716

Values represent means ± standard error of the mean (*n* = 3 animals per tank; *n* = 3 tanks by treatments). ^a,b^ Different letters indicate statistical differences between experimental diets by Tukey’s test (*p* < 0.05). *, ** Symbol indicates statistical differences within the same diet at different temperatures by Tukey’s test (*p* < 0.05). T represents *p*-value of temperature; D represents *p*-value of diet; and DxT represents *p*-value of diet versus temperature. ∑SFA, ∑MUFA, ∑PUFA, ∑LC-PUFA, ∑n-3 PUFA, and ∑n-6 PUFA are the sum of saturated (SFA), monosaturated (MUFA), polyunsaturated (PUFA), long-chain polyunsaturated fatty acids (LC-PUFA), polyunsaturated n3 (n-3 PUFA), and polyunsaturated n6 (n-6 PUFA), respectively. DHA/EPA is the ratio between the sum of total DHA (docosahexaenoic acid, 22:6n-3) and EPA (eicosapentaenoic acid, 20:5n-3).

## Data Availability

The data presented in this study are available in the article. Additionally, histological slides are available upon request from the corresponding author.
